# Conformational Sampling of Small Molecules With iCon: Performance Assessment in Comparison With OMEGA

**DOI:** 10.3389/fchem.2018.00229

**Published:** 2018-06-19

**Authors:** Giulio Poli, Thomas Seidel, Thierry Langer

**Affiliations:** ^1^Department of Biotechnology, Chemistry and Pharmacy, University of Siena, Siena, Italy; ^2^Department of Pharmaceutical Chemistry, University of Vienna, Vienna, Austria

**Keywords:** conformer generation, conformational analysis, drug design, pharmacophore modeling, virtual screening

## Abstract

Herein we present the algorithm and performance assessment of our newly developed conformer generator iCon that was implemented in LigandScout 4.0. Two data sets of high-quality X-ray structures of drug-like small molecules originating from the Protein Data Bank (200 ligands) and the Cambridge Structural Database (481 molecules) were used to validate iCon's performance in the reproduction of experimental conformations. OpenEye's conformer generator OMEGA was subjected to the same evaluation and served as a reference software in this analysis. We tested several setting patterns in order to identify the most suitable and efficient ones for conformational sampling with iCon; equivalent settings were also tested on OMEGA in order to compare the results obtained from the two programs and better assess iCon's performance. Overall, this study proved that iCon is able to generate reliable representative conformational ensembles of drug-like small molecules, yielding results comparable to those showed by OMEGA, and thus is ready to serve as a valuable tool for computer-aided drug design.

## Introduction

Conformer generation still represents a remarkably important topic within the Computer-Aided Molecular Design (CAMD) field. The exploration of the conformational space of small molecules is a challenging task that is required for different applications ranging from the search for the molecule conformation at its global energy minimum to the generation of conformational ensembles that properly represent all possible low-energy spatial dispositions that molecules are allowed to assume. Particularly, this latter analysis constitutes a fundamental step in many *in-silico* studies comprising pharmacophore modeling and pharmacophore-based virtual screening (VS) (Güner et al., 2004; Wolber and Langer, 2005), shape-based similarity searches (Hawkins et al., 2007; Sastry et al., 2011), docking and other VS methods (Cross et al., 2010; McGann, 2012), as well as different approaches like 3D and 4D QSAR modeling (Shim and MacKerell, 2011). Moreover, these techniques require different levels and qualities of conformational sampling depending on the specific goals they aim at. Therefore, it is important to balance speed and thoroughness of the conformational sampling process depending on the size of the database to be screened, in order to produce an appropriate conformational ensemble size that still guarantees reliable results. In this context, automated clustering algorithms have been recently applied for resampling conformational ensembles of small molecules (Kim et al., 2017). Such clustering approaches based on RMSD matrices, which can also find application in post-processing docking results (Tuccinardi et al., 2014), were employed to filter out unrepresentative molecular conformers with the aim of reducing the size of ensembles and data, but still providing a high coverage of the ligand's conformational space. These data highlight that the conformational sampling of small molecules is still a hot topic in CAMD. Due to the different tasks conformer generators are asked for, it is not surprising that a substantial number of programs based on different sampling algorithms (Hawkins, 2017) belonging to both stochastic (Chang et al., 1989; Treasurywala et al., 1996; Saunders, 1998; Güner et al., 2004; Watts et al., 2010) and deterministic methods (Smelliem et al., 2003; Renner et al., 2006; Li et al., 2007; Hawkins et al., 2010) have already been developed. In particular, deterministic sampling algorithms are used within several well-known conformer generators employed for VS application, including CONAN (Smelliem et al., 2003), ROTATE (Renner et al., 2006), CAESAR (Li et al., 2007), and OMEGA (Hawkins et al., 2010; OpenEye Scientific Software, 2013) whose performance has been widely validated and compared to other software (Boström, 2001; Good and Cheney, 2003; Loferer et al., 2007; Schwab, 2010; Friedrich et al., 2017). Anyway, novel software is continuously appearing on the CAMD scene, where always newer and more efficient tools are needed, and they are tested for their ability of mapping the conformational space and reproducing the conformations of experimentally determined crystal structures of drug-like small molecules (Miteva et al., 2010; O'Boyle et al., 2011; Ebejer et al., 2012; Friedrich et al., 2017). Here we report the algorithm and the performance assessment of the novel conformer generator iCon implemented in LigandScout (Wolber and Langer, 2005) which uses a systematic, knowledge-based approach for the generation of conformational ensembles to be employed in the generation of pharmacophore models and in the creation of screening databases for pharmacophore-based searches. With the aim of best analyzing iCon's performance, we evaluated representative data sets of test compounds to be used in our study. Recently, Hawkins and co-workers reported an algorithm validation of the conformer generator OMEGA for its default settings by using two sets of high-quality crystallographic structures of small molecules originating from the Protein Data Bank (PDB) (Berman et al., 2000) and the Cambridge Structural Database (CSD) (Allen, 2002) that were selected by filtering larger data sets used in previous studies (Hawkins et al., 2010). These data sets were then further refined after an analysis aimed at better understanding their suitability for conformational sampling (CS) validation as well as identifying and studying OMEGA's failures, showing that they were able to well represent the torsion angle space of the parent sets (Hawkins and Nicholls, 2012). Stimulated by these analyses, we decided to use these data sets to validate the performance of iCon regarding the reproduction of crystallographic conformations of drug-like small molecules and to compare it to the corresponding results obtained with OMEGA. A wide panel of different settings has been tested for iCon in the attempt to identify the most suitable ones. In particular, we analyzed the impact of the main conformational sampling parameters on the size and quality of the conformational ensembles generated by iCon for the two data sets of small molecules using 20 different setting patterns. For each setting, the reliability of the conformers generated by iCon for the test ligands was evaluated based on the accuracy in the reproduction of their experimental conformations, which was assessed by using two different metrics of conformational similarity. The same analysis was also performed using the software OMEGA, which is one the best conformer generators available today and thus served as the reference for iCon's performance evaluation. The quality of the conformational ensembles generated with OMEGA using 20 setting patterns corresponding to those tested with iCon was assessed and the results produced by the two software packages were compared. Based on the whole analysis, the reliability of the new conformer generator iCon was demonstrated and the most suitable iCon's setting patterns were identified.

## Materials and methods

### Data sets preparation

Two different data sets comprising 200 X-ray ligand structures originating from the Protein Data Bank and 481 X-ray structures from the Cambridge Structural Database, representing the final data sets of structures used by Hawkins and co-workers in their reported analyses concerning OMEGA's performance (Hawkins and Nicholls, 2012), were employed in this study.

For the creation of the PDB data set, we analyzed the PDB complexes from which the ligands used in Hawkin's study were extracted (see Supplementary Material) to obtain the corresponding ligand three-letter codes. The structures of all ligands were downloaded from the RCSB Ligand Expo database (www.ligand-expo.rcsb.org) in sd-file format. Hydrogen atoms were added to the ligands by using LigandScout 4.0 (Inte:Ligand GmbH, 2015) and then the molecules were visually checked for correctness on the basis of their corresponding parent X-ray complexes. For the creation of the CSD data set, the list of CSD molecules used in Hawkin's study was directly downloaded from the CSD database (in sd-file format). The so obtained experimental ligand conformations served as reference structures in the computation of root mean square deviation (RMSD) and Tanimoto Combo (TC) score values for the corresponding conformers generated by iCon and OMEGA (vide infra). To avoid any bias that could affect the conformer generation by starting from 3D structures, the two data sets were converted into SMILES notation by using OpenEye's Babel 3.327 (OpenEye Scientific Software, 2010). The obtained 681 SMILES codes eventually served as the input for iCon and OMEGA and could be processed without any issues by the two programs.

### Conformer generation with iCon

**Figure 1:**
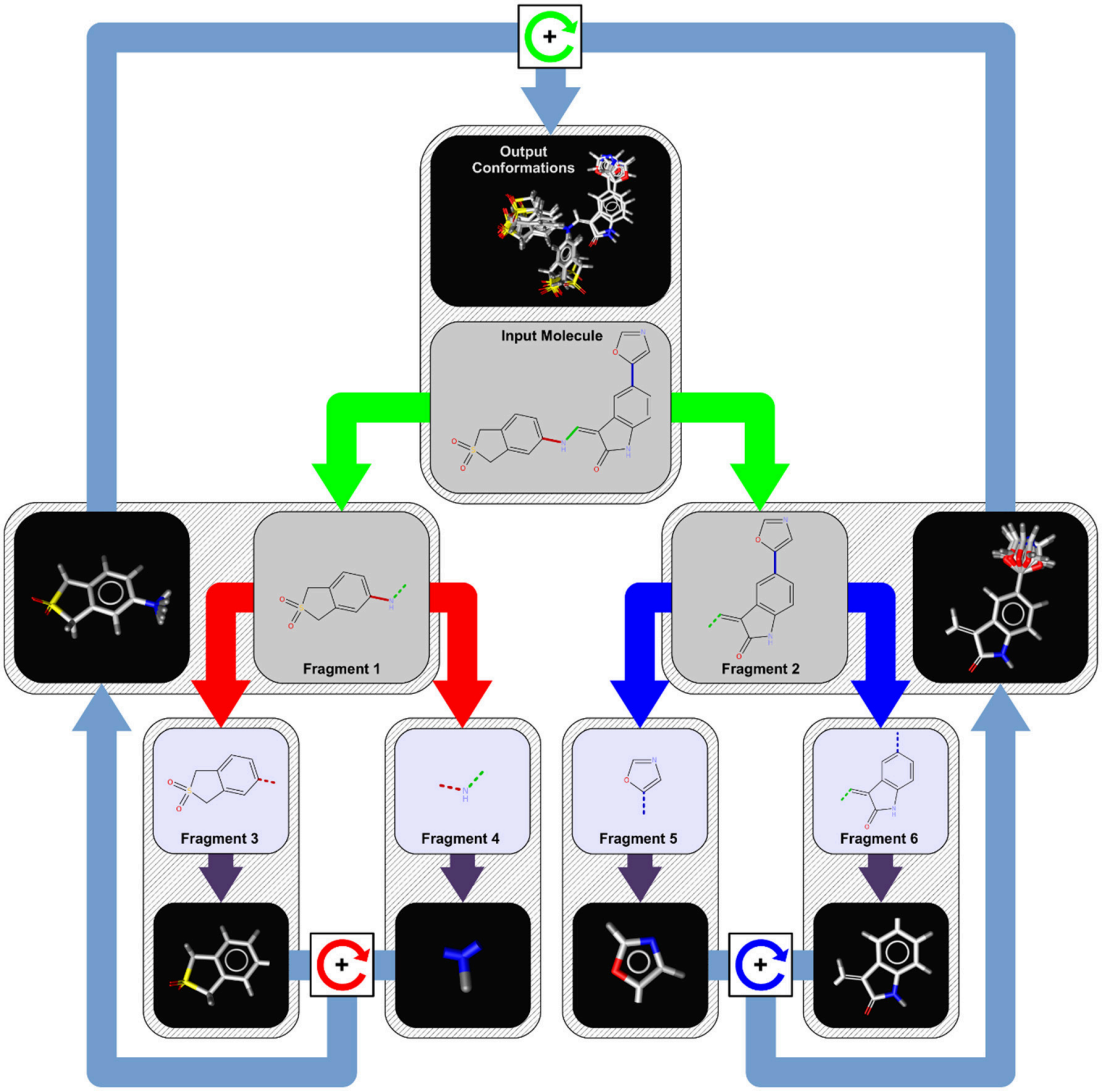
Conformer generation workflow for an example molecule with three rotatable bonds. In the first step, the input molecule is dissected recursively at each of its rotatable bonds (marked in red, green, and blue) into a tree-like structure of fragments (tree nodes) of approximately equal structural complexity. Fragments at the leaf nodes (fragments 3, 4, 5, and 6) represent the smallest rigid conformational units of the input molecule (like ring systems, atoms in chains, etc.) and are assigned initial 3D coordinates by a distance geometry/force field optimization approach (purple arrows). To generate conformer ensembles for flexible fragments (fragment 1 and 2), the coordinates of the two leaf fragments are combined by relative rotations around the connecting rotatable bond (boxes with circular arrows) of the parent fragment using the angles provided by a bond specific (or default) torsion rule. This procedure is repeated until a final set of candidate conformations for the root node (input molecule) has been obtained from which the requested number of output conformations under the given energy window and RMSD constraints are selected.

Since OMEGA's algorithm has been broadly discussed elsewhere (Hawkins et al., 2010) here we describe the conformer generation algorithm of iCon, which uses a systematic, knowledge-based approach for the generation of conformer ensembles similar to CAESAR (Li et al., 2007). The overall process is presented schematically in Figure 1 and can be divided into four logical phases that are described in more detail below.

#### Phase 1: input molecule analysis and fragmentation

When iCon starts to process an input molecule, the first step is the perception of all the rotatable bonds within the molecule. A rotatable bond is any single bond that is not a member of a ring system and connects only non-terminal heavy atoms (e.g., a bond to a methyl group or chlorine is not considered as rotatable). For each detected rotatable bond, a lookup in the built-in torsion rule database is performed to extract preferred relative torsions that are characteristic for the substituents of the bond. If a matching torsion rule cannot be found, one of the hard-coded fallback rules is applied which provide default torsion angles depending on the hybridization state of the bonded atoms. The next step is the perception of any topological symmetry that may occur in the input molecule. The thus obtained automorphism mappings of the heavy atoms are used in the conformer build-up stage for the detection of generated duplicate conformations that need to be discarded. The last step in phase 1 is the logical transformation of the input molecule into a tree-like hierarchy of structure fragments (see Figure 1). This is done by splitting the input molecule (which represents the root node of the tree) at its most central rotatable bond (green bond) into two smaller fragments of nearly the same structural complexity (fragments 1 and 2). The same procedure is then applied recursively to the two initial fragments until only fragments that cannot be partitioned any further remain. Those terminal fragments (fragments 3, 4, 5, and 6) represent the smallest conformational units of the input molecule and can be either simple heavy atom centers (e.g., -CH2-), rigid chain fragments (e.g., >C = C <) or various kinds of ring systems and combinations thereof.

#### Phase 2: generation of terminal fragment conformations

Initial conformations assigned to the structural units at the leaf nodes of the fragment-tree serve as the primary building-blocks for the recursive assembly of fragment conformer ensembles on higher tree-levels. Conformer 3D coordinates are generated by the following procedure which is based on a distance geometry approach: First, a distance bounds matrix is generated using the connection table of the fragment. The distance constraints are then augmented by volume constraints for defined chiral centers and any planar moieties of the fragment. In the next step, random 3D coordinates are assigned to each atom and then optimized to fulfill the distance and volume constraints. The thus obtained raw coordinates are further refined using a modified version of the static Merck Molecular Force Field (MMFF94s) (Halgren, 1996a,b,c,d, 1999a,b; Halgren and Nachbar, 1996) where electrostatic interactions are not considered in the energy calculation. In the case of terminal fragments representing flexible ring systems, multiple conformations of the system may be possible. If enabled (as by default, *enum-rings* option), the geometry optimization procedure is therefore repeated many times to obtain a set of multiple unique conformations of the ring system until a maximum number of subsequently failed attempts to generate a conformations or the timeout limit (*max-frag-build-time* option) has been exceeded. Terminal fragments containing invertible nitrogen atoms are also treated specially (if enabled as by default with the *enum-nitrogens* option). For such fragments, the substituents of each invertible nitrogen atom are simply flipped and again refined in the force field to yield a second set of fragment 3D coordinates. The generation of terminal fragment conformations by the just described distance geometry/force field optimization procedure is quite simple but rather time consuming. For the speedup of the overall process, calculated terminal fragment conformations get stored in a continuously growing (up to an internal maximum size) dedicated cache. Whenever a future input molecule with an already processed fragment is encountered, the lengthy calculations can be bypassed and the cached fragment conformations are used instead.

#### Phase 3: generation of flexible fragment conformers

Phase 3 is concerned with the recursive assembly of conformer ensembles which is starting at the terminal fragments. For an explanation of the process let us consider the assembly of two fragments FX and FY at level L+1 of the tree into the larger parent fragment FXY at level L. Fragments FX and FY are connected by the rotatable bond BXY of the parent fragment and the conformations of both child fragments are available, either because assembled at a lower level or because generated in phase 2. At this stage, all conformers of FX and FY contain no duplicates, show no atom clashes, satisfy the user specified energy window constraint (*e-window* option) and are ordered by increasing MMFF94 energy. The assembly of FX and FY comprises the following sub-steps: The first step is to align the bond BXY in all conformers of both FX and FY in a way that the bond has the same standard orientation (e.g., in direction of the x-axis). In the next step, a conformation from FX and one from FY is selected and their coordinates are combined with a relative torsion angle taken from the list of favorable torsions provided by the assigned torsion library entry. Afterwards, the MMFF94 energy of the new conformer candidate is calculated and compared with the energies of the previously generated conformations. If the difference between the candidate conformation energy and the energy of the lowest energy conformer so far is larger than the user specified energy window, the new conformation gets rejected because any generated parent fragment conformations will then also exceed the energy threshold. One thing to note is that there is no explicit check for atom clashes in iCon. Conformers with Van der Waals clashes show a rather high MMFF94 energy that always exceeds the specified energy window and in turn leads to their automatic exclusion from any further processing. The next step is to make sure that the generated conformer is not a duplicate of a previously generated conformation. Duplicates may always arise due to local rotational symmetries and must be excluded from the final list of fragment conformations. If the candidate conformation is not a duplicate, it gets inserted into the list of intermediate fragment conformers. If the inserted conformation is the new lowest energy conformation found so far, any previously generated conformations that now exceed the energy window are discarded. The number of fragment conformers stored at each node has an upper limit and is calculated dynamically depending on the number of rotatable bonds, the number of requested output conformations and the tree level. For the root node the limit is set to *max(PS, 5*×*N)* where *N* is the number of requested output conformation (*max-num-confs* option) and PS is the value of the *max-pool-size* option. For an internal node the maximum ensemble size depends on the number of rotatable bonds in the subtree and on the number of requested conformers of the parent node. If the maximum ensemble size is exceeded by a new conformation, the highest energy conformer is simply discarded to keep the ensemble size at its upper limit.

#### Phase 4: selection of output conformations

Once a pool of candidate low energy conformations of the input molecule has been obtained, the requested number of output conformations is selected under the specified RMSD constraints (*rms-thresh* option). The selection algorithm works as follows: First, the list of root fragment conformations is ordered by increasing MMFF94 energy value and the lowest energy conformer is put into the list of output conformations. Using this conformer as a reference, the list of fragment conformations is searched in order of increasing energy to find a new conformer whose heavy atom 3D coordinates differ at least by the specified RMSD threshold. If such a conformation could be found, it is put into the list of output conformers and the search for the next sufficiently different conformation continues. This process is repeated until the requested number of output conformations or the end of the list of fragment conformations has been reached.

### Conformational model generation

A total of 20 different setting patterns was used for the generation of conformational models of the two compound collections (Table 1). In each setting pattern, three parameters that have an analogous meaning in iCon and OMEGA (version 2.4.6.35) were systematically modified, while all other parameters were left unchanged. The parameters modified in the different settings are: *e-window, max-num-conf*, *rms-thresh* in iCon and *ewindow, maxconfs, rms* in OMEGA. The *e-window* and *ewindow* parameters define the strain energy window allowed for conformers to be included in the final ensemble of conformers. Conformers with strain energy higher than the sum of the energy of the global minimum conformer and the *e-window*/*ewindow* value are rejected. The default *ewindow* value for OMEGA is 10 kcal/mol. The *max-num-conf* and *maxconfs* parameters define the maximum number of conformers that can be included in the final ensemble of conformers (the default *maxconfs* setting for OMEGA is 200). If the number of conformers satisfying the energetic criteria is higher than the allowed limit, conformers with the highest strain energies are rejected until the threshold value is reached. *Rms-thresh* and *rms* parameters define the minimum RMSD of coordinates below which two conformers are considered as duplicates. The default value for OMEGA's *rms* option is 0.5 Å. To simplify the analysis of results, the settings patterns were divided in low, medium, and high accuracy settings depending on the average number of conformers (NOC) generated for the compounds of the PDB data set: up to 100 for low accuracy settings, from 100 to 200 for medium accuracy settings and from 200 to 500 for high accuracy settings.

**Table 1:** Setting patterns tested for conformer generation with iCon and OMEGA.

**Setting name**	***MC^a^***	***EW^b^* (kcal/mol)**	***RT^c^* (Å)**
LowAcc_1	25	10	0.8
LowAcc_2	25	15	0.5
LowAcc_3	50	10	0.5
LowAcc_4	50	15	0.5
LowAcc_5	100	15	0.8
LowAcc_6	100	15	0.5
LowAcc_7	200	20	0.8
MedAcc_1	200	10	0.5
MedAcc_2	200	15	0.5
MedAcc_3	200	20	0.5
MedAcc_4	350	15	0.5
MedAcc_5	350	20	0.5
MedAcc_6	400	25	0.8
HighAcc_1	400	25	0.5
HighAcc_2	500	20	0.5
HighAcc_3	500	25	0.5
HighAcc_4	500	25	0.2
HighAcc_5	800	30	0.5
HighAcc_6	800	35	0.5
HighAcc_7	800	25	0.2

a *MC, max-num-conf/maxconfs*;

b *EW, e-window/ewindow*;

c *RT, rms-thresh/rms*.

### Computation of RMSD values

RMSD values between the experimental ligand conformations and the related ensembles of conformers generated by iCon and OMEGA employing the different setting patterns were calculated for each molecule. Only heavy atoms were considered in the RMSD computation, without including any mass-weighted term. For each molecule only the RMSD value between the crystallographic conformation and the best-fitting conformer was considered for performance analyses. For the actual calculation of the heavy atom RMSD of two conformations an alignment in 3D space is required. The alignment was performed by a Java implementation of Kabsch's algorithm (Kabsch, 1976, 1978) which calculates the optimal rotation matrix that minimizes the RMSD between two paired sets of points (positions of the heavy atoms). Rotational symmetries were considered in the alignment and RMSD calculation by trying all possible pairings of equivalent heavy atoms and then using only the lowest obtained RMSD for the comparison of the two conformations.

### Computation of tanimoto combo scores

The Tanimoto Combo (TC) represents a complementary metric with respect to the RMSD to compare experimental and generated ligand conformations. It comprises two different scores: shape Tanimoto and color Tanimoto. Shape Tanimoto refers to the structural shape similarity whereas color Tanimoto refers to the matching of the ligands functional groups. Each score provides a contribution ranging from 0 to 1 to the TC score, which can thus assume values between 0 and 2. The TC score relative to the superposition between the experimental conformations of the test compounds and the related ensembles of conformers generated by iCon and OMEGA were calculated by using the Shape Toolkit (Haigh et al., 2005) implemented in ROCS (Hawkins et al., 2007; OpenEye Scientific Software, 2012) from OpenEye Scientific Software. Shell scripts were employed to allow the automated calculation of the TC score values for the conformer ensembles generated with the different tested setting patterns. For each compound, only the TC score of the superposition between the crystallographic conformation and the best-matching generated conformer was used for performance analyses.

### Hardware specifications

All calculations considering computation time were performed on a single Intel i7-3770K 3.50 GHz PC equipped with 8 GB RAM running Linux Centos 5.8. All calculations were done in single CPU mode.

## Results and discussion

In order to evaluate the performance of iCon in reproducing experimentally determined ligand conformations, two data sets of high quality X-ray structures originating from the PDB and CSD were created. These data sets comprise a total of 681 structures (200 for the PDB data set and 481 for the CSD data set) and were selected by Hawkins and co-workers to validate the performance of their conformer generator OMEGA (Hawkins and Nicholls, 2012). The choice of these structures as test set for iCon's validation was also driven by the intention to use OMEGA as a reference software, since it is one of the best conformer generators available today.

### Data set properties

**Figure 2:**
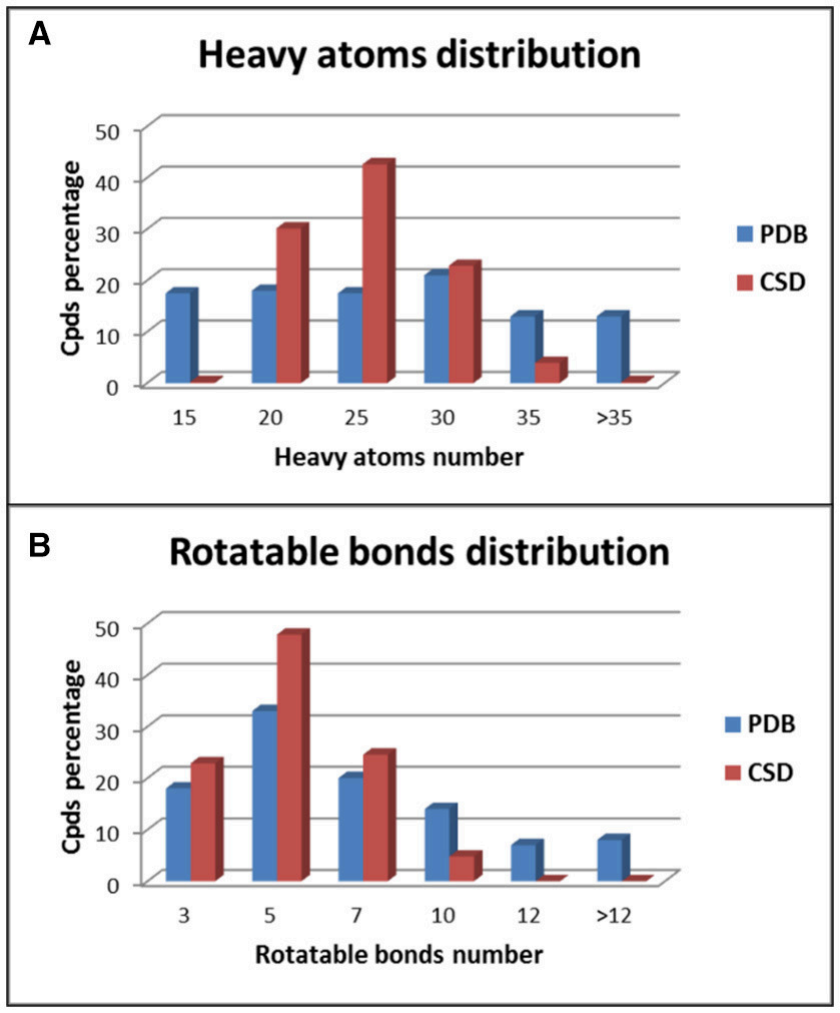
Distribution of heavy atoms **(A)** and rotatable bonds **(B)** among PDB and CSD compounds.

The two data sets show different distributions of heavy atoms and rotatable bonds among the test compounds. For the ligands belonging to the PDB data set a quite homogeneous distribution of the heavy atoms (HAs) was observed, especially for compounds with up to 30 HAs (Figure 2A). On the contrary, about 95% of CSD compounds showed a number of HAs ranging from 15 to 30 and in particular almost 45% of molecules presented 21–25 HAs. Regarding the distribution of the number of rotatable bonds (RBs) in the data set compounds, PDB ligands showed again a more homogeneous trend with respect to the CSD structures (Figure 2B). In the CSD data set about 95% of compounds had less than 7 RBs and no molecules with more than 9 rotors were found, whereas 29% of PDB ligands presented more than 7 RBs and 15% of compounds showed an average of 13 rotors. All these data indicate that the PDB data set comprises molecules with a larger range of molecular weight compared to the CSD structures and with a higher conformational freedom. This makes the conformations of PDB ligands more challenging to reproduce with respect to the CSD molecules.

### Influence of the sampling parameters on the NOC

The NOC generated by conformational sampling strongly influences the performance of a conformer generator in reproducing experimentally derived conformations; the higher the NOC in a conformational ensemble, the higher the probability that a conformer well-fitting the experimental one can be found in that ensemble. On the other hand, the quality of the sampling process also depends on the way the conformational space of the molecules is sampled. For example, the generation of an elevated number of redundant conformers does not help in the exploration of all the dispositions that a molecule can assume according to its conformational freedom, but increases the calculation time and the data file size. This is the reason why the different parameters influencing the NOC should be reciprocally calibrated, so that the compounds conformational space can be adequately covered according to the NOC generated through the sampling process. To this aim, understanding how the different parameters affect the conformational sampling of different compounds is an important issue.

**Figure 3:**
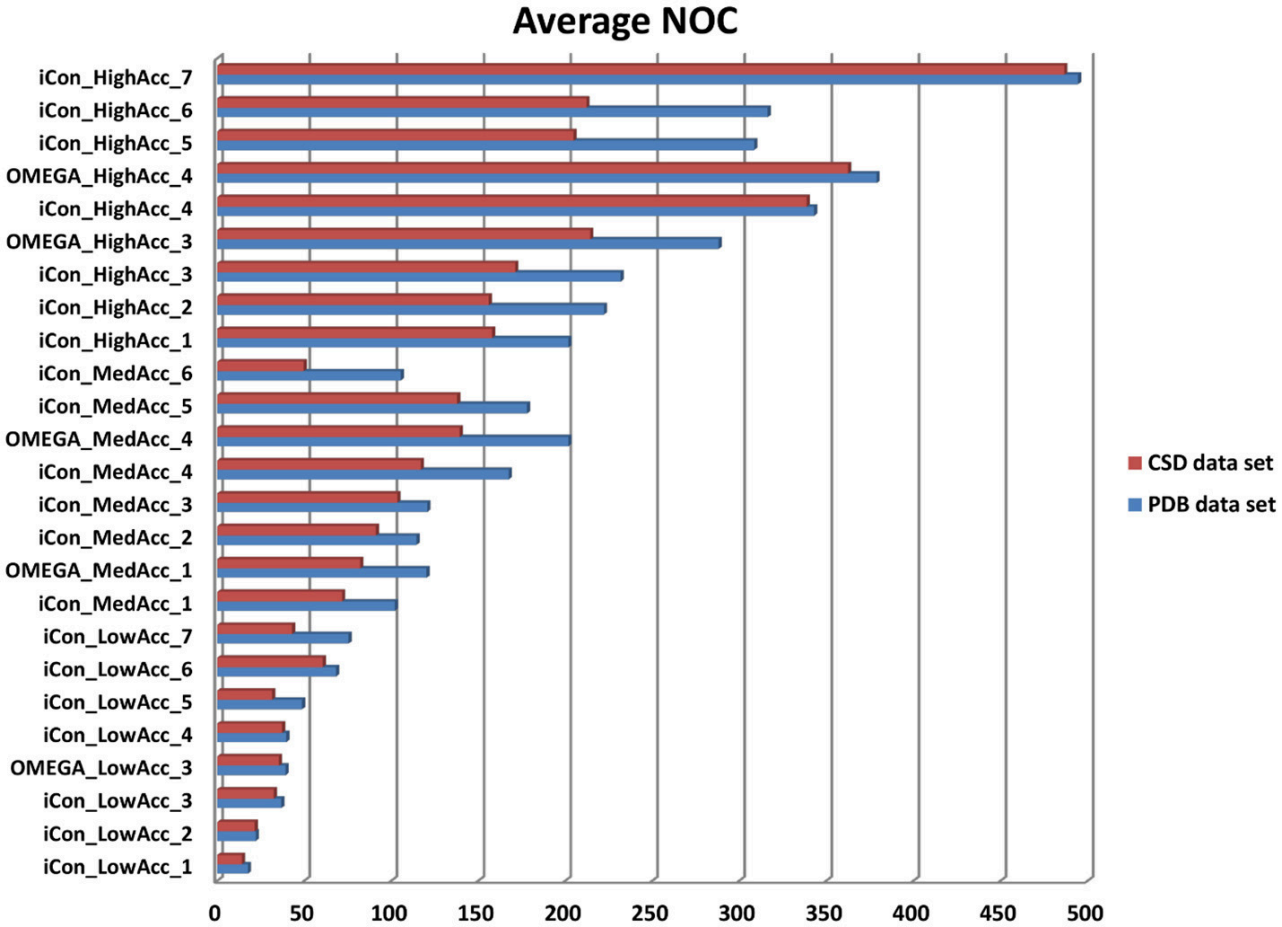
Number of conformers (NOC) generated by iCon and OMEGA for PDB data set (blue bars) and CSD data set compounds (red bars) with different settings. For iCon all the tested settings are reported; for OMEGA only some representative settings are shown as a reference.

In Figure 3 the average NOC generated with iCon for the two data sets by employing all the different setting patterns is shown, together with some of the results obtained with OMEGA by using the same settings. As expected according to the molecular properties analyzed for the two data sets, a higher NOC was always generated for the PDB ligands and this difference increased along with the maximum NOC allowed for the ensembles. This trend can be best observed by comparing the results obtained with iCon for *MedAcc_3* and *HighAcc_2* settings, differing only in the values of *max-num-conf* (200 and 500, respectively). In fact, the difference between the average NOC produced for CSD and PDB compounds increased more than 3.5 times passing from *MedAcc_3* to *HighAcc_2*. Therefore, the *max-num-conf* parameter showed to have a stronger influence on conformer generation for PDB ligands than for CSD compounds.

Conversely, when the NOC was increased due to a lower RMSD threshold allowed among the output conformers, the difference between the NOC for CSD and PDB compounds was found to be smaller. This is clearly shown by the comparison of *HighAcc_3* and *HighAcc_4* settings, for which a reduction of the *rms-thresh* value from 0.5 to 0.2 Å determined the generation of a much higher NOC for both data sets, but with a really smaller gap between them (PDB/CSD NOC with *HighAcc_3* settings = 232/171; PDB/CSD NOC with *HighAcc_4* settings = 343/339). Interestingly, increasing the *max-num-conf* value up to 800 in the *HighAcc_7* settings raised the NOC produced for the two data sets to almost 500 conformers per ensemble although maintaining such a small gap. These findings indicate that both *max-num-conf* and *rms-thresh* parameters have a strong influence on the NOC. Anyway, for compounds with less conformational freedom a low RMSD threshold has a bigger impact for the production of large conformational ensembles, even though it can lead to the generation of too similar conformers.

The value for the energy window seemed to have a lesser effect than the other two parameters on the NOC generated for the PDB ligands. Raising the *e-window* from 10 to 15 and 20 kcal/mol without changing *max-num-conf* and *rms-thresh* values (passing from *MedAcc_1* to *MedAcc_2* and *MedAcc_3* settings, respectively) produced a 12 and 18% increase in the NOC, respectively. Nevertheless, the energy window appears to have a greater influence on the size of the conformational ensembles produced for the CSD compounds, as the same changes resulted in a 27 and a 44% increase in the NOC for these molecules.

All the considerations reported above are also valid for OMEGA as the same trends relative to the variations of the NOC generated for the two data sets are observed. OMEGA always produced a higher NOC than iCon for all tested setting patterns, especially for high accuracy settings (on average a 9.3, 20.1, and 24.0% higher NOC for low, medium and high accuracy settings, respectively), with a corresponding wider gap between the NOC generated for the CSD and PDB compounds (see also Supplementary Figure 1).

### Influence of rotors on the NOC

**Figure 4:**
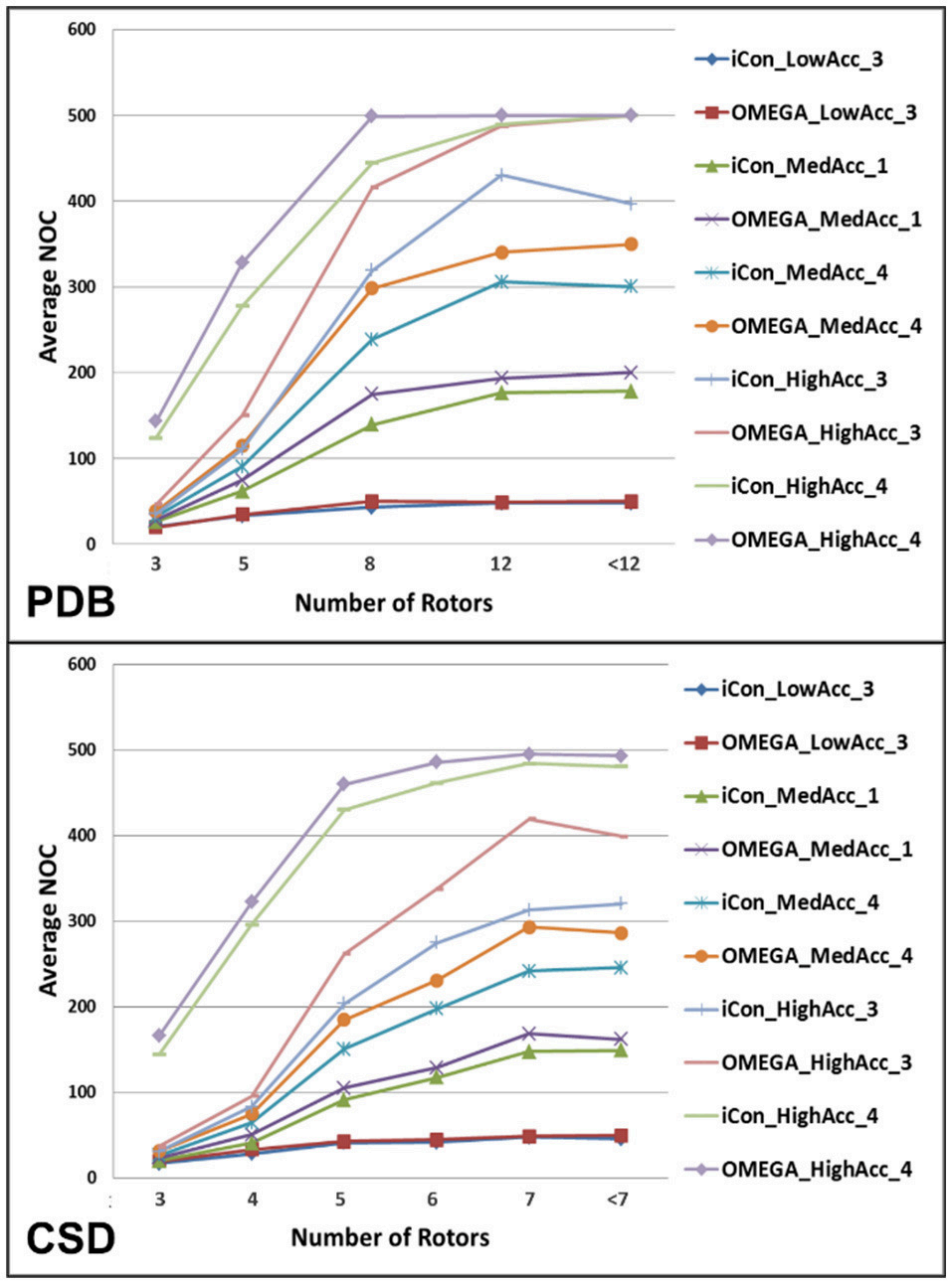
Average NOC generated by iCon and OMEGA, for some representative setting patterns, as a function of the number of rotatable bonds of PDB and CSD compounds. Due to the different rotor distribution in PDB and CSD molecules, different scales have been considered for the two data sets.

The analysis of the variation of the average NOC as a function of the number of rotatable bonds (RBs) clearly highlighted the unsurprisingly strong dependence of the conformer generation process on the conformational freedom of the compounds. For molecules with 3 or less rotors, ensembles of up to 50 conformers were generated for all the tested settings except for those where the RMSD cutoff was set to 0.2 Å, which produced nearly a three-fold higher NOC (Figure 4). For ligands with 8 or more rotors ensembles comprising a minimum of 120 conformers (up to several hundreds) were generated for medium and high accuracy settings, where a wider conformational variability was allowed in the sampling process (see Supplementary Figure 2). As shown in Figure 4, the two conformer generators presented a similar trend in the NOC generated for the analyzed compounds with respect to their number of RBs. However, the increase in the number of rotors produced a slightly steeper increase in the NOC generated by OMEGA. This became even more evident when settings patterns producing high average NOC were considered. Anyway, setting the RMSD threshold to 0.2 Å reduced this difference, as shown by the comparison of the NOC generated with *HighAcc_3* and *HighAcc_4* settings.

### Performance assessment

The ability of the software iCon to reproduce the crystallographic conformation of the data set compounds was studied by using two different metrics: the root mean square deviation (RMSD) and the Tanimoto combo (TC) score, which were calculated for the generated ligand conformers using the corresponding experimental conformation as reference. These analyses were carried out on the conformers generated by using all the 20 different settings patterns reported in Table 1. Only the values for the best-fitting conformers were taken into consideration, i.e., the lowest RMSD and the highest TC score obtained for each ligand conformational ensemble. In the same way the conformers generated by OMEGA using equivalent settings were analyzed, in order to compare the performance of the two software packages. To get a global overview of iCon's performance as a function of the various settings used and to compare it to OMEGA's performance, we calculated the average values of RMSD and TC scores obtained for the best-fitting conformers of the PDB and CSD compounds. Additionally, the number of ligands giving a RMSD value higher than 2 Å (RMSD failures) and the number of ligands giving a TC score lower than 1 (TC failures) were also reported and used as a secondary metric for performance assessment and comparison. The results obtained by applying low, medium, and high accuracy settings for the conformer generation of PDB and CSD ligands are reported in Tables 2–4, respectively. As expected, the PDB data set showed to be more challenging than the CSD data set, since for the CSD compounds both conformer generators gave significantly better RMSD and TC score values with respect to those produced for the PDB ligands. Accordingly, the number of RMSD and TC failures yielded by the two programs for the CSD data set were consistently lower than those reported for the PDB data set, which in fact contained a higher percentage of large compounds with a higher conformational freedom (see section Data Set Properties).

**Table 2:** Mean RMSD and TC score values obtained for PDB and CSD data set compounds by using iCon and OMEGA with low accuracy settings.

	**Settings**	**LA^a^_1**	**LA_2**	**LA_3**	**LA_4**	**LA_5**	**LA_6**	**LA_7**
	**Parameters**	MC^b^ = 25	MC = 25	MC = 50	MC = 50	MC = 100	MC = 100	MC = 200
		EW^c^ = 10	EW = 15	EW = 10	EW = 15	EW = 15	EW = 15	EW = 20
		RT^d^ = 0.8	RT = 0.5	RT = 0.5	RT = 0.5	RT = 0.8	RT = 0.5	RT = 0.8
**iCon**	PDB mean RMSD	0.96	1.00	0.91	0.89	0.84	0.84	0.81
	PDB RMSD failures	12	18	13	11	4	7	4
	PDB mean TC	1.41	1.40	1.44	1.45	1.47	1.48	1.49
	PDB TC failures	25	32	25	24	16	19	14
	CSD mean RMSD	0.63	0.59	0.55	0.52	0.56	0.49	0.54
	CSD RMSD failures	1	2	1	1	0	1	0
	CSD mean TC	1.64	1.68	1.69	1.72	1.69	1.74	1.71
	CSD TC failures	4	7	5	3	1	1	0
**OMEGA**	PDB mean RMSD	0.94	0.97	0.90	0.89	0.82	0.80	0.78
	PDB RMSD failures	11	15	12	12	5	6	3
	PDB mean TC	1.43	1.45	1.48	1.49	1.50	1.53	1.52
	PDB TC failures	27	29	26	26	12	20	7
	CSD mean RMSD	0.64	0.61	0.56	0.54	0.57	0.50	0.54
	CSD RMSD failures	4	7	3	3	2	3	0
	CSD mean TC	1.63	1.66	1.69	1.71	1.68	1.73	1.70
	CSD TC failures	8	12	5	4	3	3	0

a *LA, LowAcc*;

b *MC, max-num-conf/maxconfs*;

c *EW, e-window/ewindow*;

d *RT, rms-thresh/rms*.

### Influence of the sampling parameters on iCon's performance

The influence of the sampling parameters on iCon's performance was in agreement with their effect on the NOC generated. The *max-num-conf* parameter showed the strongest impact on the quality of the conformational sampling outcome when low accuracy settings were used. In this case, the maximum number of conformers allowed was quite small and represented the main limit to the generation of larger ensembles and to sampling accuracy. The increase of *max-num-conf* from 25 in *LowAcc_2* to 50 in *LowAcc_4* settings gave a difference in mean RMSD and TC score values of −11.0 and +3.57%, respectively, for PDB ligands, while a difference of −11.86 and +2.38%, respectively, was obtained for CSD compounds (Table 2). Moreover, this settings change produced a strong reduction of the number of failures for both data sets (from −25% up to −70%). This suggested that a *max-num-conf* value lower than 50 is too restrictive even for the generation of small ensembles, rejecting valuable conformers for an adequate sampling of the molecule's conformational space. By doubling again the *max-num-conf* value in *LowAcc_6* settings a lower (although still substantial) improvement in performance was obtained, in terms of both mean RMSD (−5.62% for PDB and −5.77% for CSD compounds) and TC score values (+2.07% for PDB and +1.26% for CSD data set). Finally, passing from *MedAcc_3* (*max-num-conf* = 200, Table 3) to *HighAcc_2* settings (*max-num-conf* = 500, Table 4) even smaller improvements were obtained for both PDB (−3.95% in mean RMSD and +1.99% in mean TC score) and CSD compounds (−4.35% in mean RMSD and +0.57% in mean TC score).

**Table 3:** Mean RMSD and TC score values obtained for PDB and CSD data set compounds by using iCon and OMEGA with medium accuracy settings.

	**Settings**	**MA^a^_1**	**MA_2**	**MA_3**	**MA_4**	**MA_5**	**MA_6**
	**Parameters**	MC^b^ = 200	MC = 200	MC = 200	MC = 350	MC = 350	MC = 400
		EW^c^ = 10	EW = 15	EW = 20	EW = 15	EW = 20	EW = 25
		RT^d^ = 0.5	RT = 0.5	RT = 0.5	RT = 0.5	RT = 0.5	RT = 0.8
**iCon**	PDB mean RMSD	0.82	0.78	0.76	0.75	0.74	0.79
	PDB RMSD failures	5	4	4	3	3	2
	PDB mean TC	1.49	1.51	1.51	1.52	1.53	1.50
	PDB TC failures	15	14	15	13	14	13
	CSD mean RMSD	0.51	0.47	0.46	0.46	0.44	0.54
	CSD RMSD failures	0	0	0	0	0	0
	CSD mean TC	1.72	1.75	1.76	1.76	1.77	1.71
	CSD TC failures	3	1	0	1	0	0
**OMEGA**	PDB mean RMSD	0.76	0.75	0.75	0.72	0.72	0.76
	PDB RMSD failures	4	5	5	5	5	2
	PDB mean TC	1.56	1.57	1.57	1.58	1.58	1.54
	PDB TC failures	10	10	10	8	8	4
	CSD mean RMSD	0.51	0.48	0.45	0.47	0.44	0.53
	CSD RMSD failures	3	3	1	2	0	0
	CSD mean TC	1.73	1.75	1.76	1.76	1.77	1.71
	CSD TC failures	4	2	1	1	0	0

a *MA, MedAcc*;

b *MC, max-num-conf/maxconfs*;

c *EW, e-window/ewindow*;

d *RT, rms-thresh/rms*.

**Table 4:** Mean RMSD and TC score values obtained for PDB and CSD data set compounds by using iCon and OMEGA with high accuracy settings.

	**Settings**	**HA^a^_1**	**HA_2**	**HA_3**	**HA_4**	**HA_5**	**HA_6**	**HA_7**
	**Parameters**	MC^b^ = 400	MC = 500	MC = 500	MC = 500	MC = 800	MC = 800	MC = 800
		EW^c^ = 25	EW = 20	EW = 25	EW = 25	EW = 30	EW = 35	EW = 25
		RT^d^ = 0.5	RT = 0.5	RT = 0.5	RT = 0.2	RT = 0.5	RT = 0.5	RT = 0.2
**iCon**	PDB mean RMSD	0.73	0.73	0.72	0.75	0.71	0.70	0.72
	PDB RMSD failures	2	3	2	5	2	2	4
	PDB mean TC	1.54	1.54	1.54	1.55	1.56	1.56	1.56
	PDB TC failures	13	13	12	14	11	11	12
	CSD mean RMSD	0.44	0.44	0.44	0.40	0.43	0.43	0.39
	CSD RMSD failures	0	0	0	1	0	0	0
	CSD mean TC	1.78	1.77	1.78	1.80	1.78	1.78	1.80
	CSD TC failures	0	0	0	0	0	0	0
**OMEGA**	PDB mean RMSD	0.71	0.71	0.71	0.72	0.68	0.68	0.69
	PDB RMSD failures	3	3	3	6	2	2	5
	PDB mean TC	1.59	1.59	1.60	1.60	1.61	1.61	1.62
	PDB TC failures	7	7	7	11	5	5	9
	CSD mean RMSD	0.43	0.44	0.43	0.40	0.41	0.41	0.39
	CSD RMSD failures	0	0	0	1	0	0	1
	CSD mean TC	1.78	1.77	1.78	1.80	1.79	1.79	1.81
	CSD TC failures	0	0	0	1	0	0	1

a *HA, HighAcc*;

b *MC, max-num-conf/maxconfs*;

c *EW, e-window/ewindow*;

d *RT, rms-thresh/rms*.

An *e-window* value of 10 kcal/mol (OMEGA's default *ewindow* value) seemed to be too restrictive for iCon, since an increase of 5 kcal/mol lead to a substantial improvement in the quality of the conformational ensembles generated with *MedAcc_2* respect to *MedAcc_1* settings (Table 3), especially for the CSD data set. With *MedAcc_2* settings iCon gave a mean RMSD of 0.47 Å and a mean TC score of 1.75 for the CSD data set (−7.84% and +1.74% compared to the results obtained with *MedAcc_1* setting), while for the PDB data set mean RMSD and TC score values of 0.78 Å (−4.88%) and 1.71 (+1.34%) were obtained. This was in agreement with the deeper influence produced by this parameter on the NOC generated for CSD compounds with respect to PDB ligands (see section Influence of the Sampling Parameters on the NOC). The better results obtained with the *LowAcc_4* settings in comparison with *LowAcc_3* (Table 2), particularly for the CSD compounds (−5.45% of mean RMSD and +1.78% of mean TC score), suggested that an *e-window* of 15 kcal/mol might be also suitable for the generation of small conformational ensembles (depending on the molecular properties of the compounds to be sampled), even if at the price of a slightly higher calculation time. A further increase of *e-window* up to 20 kcal/mol was considered more appropriate for larger ensembles, since when used in *MedAcc_3* settings it did not seem to be worth the higher costs in machine time (see section Computational Resources) in light of the small improvements obtained in terms of mean RMSD and TC scores with respect to the *MedAcc_2* settings (Table 3).

As far as the *rms-thresh* value is concerned, it showed to have a quite different impact on the results obtained for the two different data sets. For the generation of small conformational ensembles an *rms-thresh* value of 0.8 Å allowed a substantial reduction of both RMSD and TC failures obtained for PDB ligands with *LowAcc_5* settings with respect to *LowAcc_6* (−43 and −26%, respectively), although accompanied by a marginal reduction of the mean TC score (Table 2). On the contrary, the results obtained for CSD compounds with *LowAcc_5* setting were considerably worse compared to those given by *LowAcc_6* (mean RMSD = 0.56 Å, +14.29%; mean TC score = 1.69, −2.87%). When higher *max-num-conf* and *e-window* values were used, a RMSD cutoff of 0.8 Å had a more deleterious effect on the size and quality of the conformational ensembles generated for CSD compounds especially in terms of mean RMSD values, for which an increment of 22.73% was obtained passing from *HighAcc_1* (mean RMSD = 0.44 Å, Table 4) to *MedAcc_6* settings (mean RMSD = 0.54 Å, Table 3). This change gave worse results also for the PDB data set (mean RMSD = 0.79 Å, +8.22%; mean TC score = 1.50, −2.60%), although without affecting the number of failures. Finally, reducing the *rms-thresh* value to 0.2 Å for the generation of very large conformational ensembles produced improvements in the results relative to the CSD data set, as observed for *HighAcc_3* and *HighAcc_4* settings (Table 4), which gave mean RMSD and TC score values of 0.40 Å and 1.80, respectively (−9.09% and +1.12%, compared to the *HighAcc_3* values). For the PDB data set, instead, this settings change seemed to result in the generation of ensembles comprising too similar conformers (with the consequent rejection of valuable ones, for some compounds), since it produced a higher number of failures and a higher mean RMSD value (0.75 Å, +4.17%), with only a marginal increase of mean TC score (1.55, +0.65%).

Taken together, these results show that in order to obtain good quality conformational ensembles, independently from the accuracy level required, it is not only necessary to reciprocally adjust the different sampling parameters, but also to calibrate them based on the molecular properties of the compounds to be sampled. Among the medium accuracy settings, *MedAcc_2* showed to be a good settings pattern for both data sets, considering the results in terms of mean RMSD and TC scores with respect to the average NOC generated, even though given the machine time required for the sampling it might not be particularly efficient for PDB-like molecules. For the same reason *LowAcc_4* seems to represent a good compromise between accuracy and computational resources for CSD compounds, but not for PDB ligands (see section Computational Resources). Nevertheless, in order to get a certain improvement in the quality of the conformational sampling of compounds with molecular properties similar to PDB ligands, a small increase of *max-num-conf* should be accompanied by a less strict RMSD cutoff (as in *LowAcc_5* setting), while for CSD-like compounds this change would only yield a negative effect. For a more exhaustive sampling the use of a lower *rms-thresh* value seemed more important than a considerable increase of *e-window* and *max-num-conf* parameters to improve the quality of conformational ensembles of CSD-like compounds. In fact, the best results for the CSD data set were obtained with *HighAcc_4* and *HighAcc_7* settings, while for PDB-like molecules, the best results were obtained by using higher *e-window* and *max-num-conf* values without reducing the RMSD cutoff (with *HighAcc_5* and *HighAcc_6* settings).

### OMEGA and iCon: overall comparison of the results for PDB and CSD data sets

**Figure 5:**
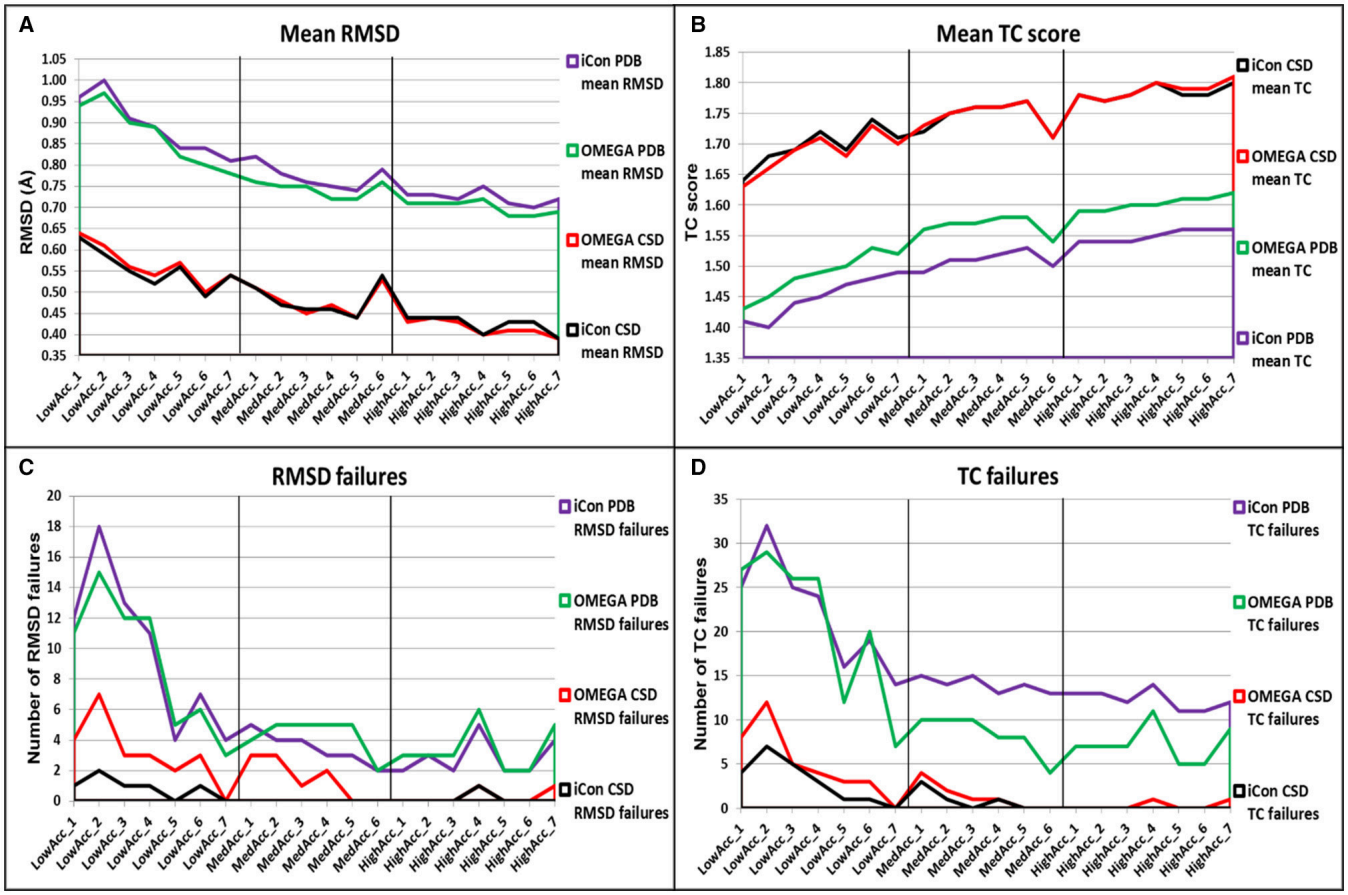
Overall comparison of **(A)** mean RMSD values, **(B)** mean TC score values, **(C)** number of RMSD failure, and **(D)** number of TC failures obtained for PDB and CSD data set compounds by using iCon and OMEGA with the different setting patterns. Black vertical lines are used to separate the three different groups of settings.

In general, despite the two conformer generators showed similar performances, OMEGA seemed to be slightly more effective in reproducing the bioactive conformation of PDB ligands, independently from the setting patterns used, since the mean RMSD values obtained with iCon were, on average, 3.23% higher than those shown by OMEGA and the TC scores were 3.11% lower (Figures 5A,B). Only with the *LowAcc_4* settings iCon showed the same mean RMSD values obtained with OMEGA. The main difference among the results obtained with the two programs for PDB data set concerned the number of TC failures, which was however significantly high for both programs when low accuracy settings were used, reaching a maximum of 16% for iCon and 14.5% for OMEGA (with *LowAcc_2* settings, Table 2). Although the average gap between the mean TC scores given by the two programs was quite small, the number of OMEGA's TC failures was about 40% lower than iCon's ones for medium and high accuracy settings (Figure 5D, Tables 3, 4). For low accuracy settings instead, the number of TC failures was comparable between the two conformer generators, with iCon giving less failures than OMEGA with 4 out of these 7 settings (Table 2). An inverse situation is observed regarding the number of RMSD failures produced by the programs. By using low accuracy settings iCon gave a lower number of RMSD failures only with *LowAcc_4* and *LowAcc_5* settings (Table 2). On the contrary, with almost all the medium and high accuracy settings the number of iCon's RMSD failures was either lower or equal to the number of OMEGA's ones (Figure 5D, Tables 3, 4).

For the CSD data set, a different trend in the performance of the two programs was observed depending on the group of setting patterns tested. As reported in Table 2, by using low accuracy settings iCon showed a slightly better performance with respect to OMEGA in terms of both mean RMSD (−2.01%, on average) and mean TC score (+0.59%, on average) values. Moreover, for these settings iCon produced a number of RMSD and TC failures corresponding, on average, to 50% of the failures shown by OMEGA (see also Figures 5C,D). By using medium accuracy settings the two conformer generators gave very similar results: the mean TC scores were practically identical and the differences in mean RMSD values minimal (Table 3, Figure 5A). Notably, for all these settings the number of iCon's RMSD and TC failures was always lower or equal to the corresponding OMEGA failures (Figures 5C,D). Finally, with high accuracy settings the difference in performance of the two conformer generators seemed almost the opposite with respect to what observed for low accuracy settings. In fact, iCon showed mean RMSD values and mean TC scores that were, on average, 2.06% higher and 0.24% lower than those obtained with OMEGA (Table 4), although it never produced a higher number of RMSD or TC failures (Figures 5C,D).

The overall comparison of iCon's and OMEGA's results showed that iCon seemed more efficient in reproducing crystallographic ligand conformations through small conformational ensembles, since by using low accuracy settings it slightly outperformed OMEGA in terms of RMSD and TC scores (and corresponding failures) for the CSD data set. Moreover, for the PDB data set, the difference in performance with respect to OMEGA, which gave slightly better results, was lower than that observed for the other groups of setting patterns. When the generation of larger ensembles was allowed as in medium and high accuracy settings, OMEGA seemed to perform relatively better than iCon, although the differences were still modest. This can be due to the facts that OMEGA always produced a NOC considerably higher than iCon for these setting patterns (see section Influence of the Sampling Parameters on the NOC) and the built-in torsion library employed by OMEGA which is biased toward PDB ligand conformations (Hawkins et al., 2010). A reasonable explanation for the in general higher NOC generated by OMEGA is the input molecule fragmentation strategy that is adopted by OMEGA. In contrast to iCon, OMEGA allows flexible terminal chain fragments (see section Conformer Generation With iCon) with multiple rotatable bonds which are in turn looked up in a built-in cache of precalculated refined fragment conformations upon overall molecule conformer assembly. This effectively reduces the number of rotatable bonds when dealing with highly flexible molecules and, as a consequence, will speed up the conformer generation process in general and also decrease the chance to produce rejected high energy conformations of the overall molecule due to steric clashes. However, it is worth noting that with medium and high accuracy settings OMEGA gave a higher number of RMSD failures for both PDB and CSD data sets and a lower number of TC failures only for PDB compounds, on average.

### OMEGA and iCon: deep comparative analysis of representative setting patterns

#### Spreading of RMSD and TC score values

For a better insight into iCon's performance and a more accurate comparison with OMEGA, we analyzed the spreading of the RMSD (Table 5) and TC score values (Table 6) that were obtained for the generated conformers of both data sets by using three representative setting patterns, one for each of the three different setting groups: *LowAcc_4, MedAcc_2*, and *HighAcc_4* (see Supplementary Tables 1, 2 for the analysis of other representative setting patterns). The results for both metrics were divided into different classes representing different levels of precision in the reproduction of the experimental conformations. A RMSD smaller than 0.5 Å, as well as a TC score higher than 1.75, correspond to an excellent matching between two different conformations, thus denoting a perfect reproduction of the compound's crystallographic pose (TC scores higher than 1.95 and RMSDs of about 0.1 Å mean conformational identity). RMSD values between 0.5 and 1.0 Å correspond to a very good matching, where all the compound's functional groups of the best-fitting generated conformers are correctly superposed to the experimental ones; the same is valid for TC scores between 1.75 and 1.50. When the RMSD lies in the 1.0–1.5 Å range and/or when the TC score is in the 1.50–1.25 range there is still a good matching between the overlaid conformations. For RMSDs between 1.5 and 2.0 Å, as well as for TC scores between 1.25 and 1.0, the representation of the crystallographic conformation is less accurate, since some of the compounds' chemical features in the generated conformers might not be correctly oriented with respect to the same moieties in the reference ligand pose, but the overall superposition is still sufficiently good. RMSDs above 2.0 Å and/or TC scores below 1.0 mean that the matching between the generated and experimental conformers is not good enough to consider the crystallographic pose as properly reproduced.

**Table 5:** Percentage spreading of RMSD values calculated for conformers of PDB and CSD compounds generated by iCon and OMEGA using three representative setting patterns.

**Setting pattern**	**Data set**	**< 0.1**	**< 0.5**	**< 1.0**	**< 1.5**	**< 2.0**	**< 3.0**	**>2.0**	**Mean**
									**RMSD (Å)**
iCon_LowAcc_4	PDB	0.0	31.5	38.0	16.5	8.5	5.0	5.5	0.89
OMEGA_LowAcc_4	PDB	0.0	25.0	48.0	12.0	9.0	5.5	6.0	0.89
iCon_LowAcc_4	CSD	0.8	61.0	29.7	6.4	1.9	0.2	0.2	0.52
OMEGA_LowAcc_4	CSD	0.2	59.3	32.8	5.8	1.3	0.6	0.6	0.54
iCon_MedAcc_2	PDB	0.0	35.5	42.5	14.0	6.0	1.5	2.0	0.78
OMEGA_MedAcc_2	PDB	0.0	32.5	48.0	11.0	6.0	2.5	2.5	0.75
iCon_MedAcc_2	CSD	0.8	68.8	25.8	3.7	0.8	0.0	0.0	0.47
OMEGA_MedAcc_2	CSD	0.2	69.0	26.4	3.3	0.4	0.6	0.6	0.48
iCon_HighAcc_4	PDB	0.0	39.5	40.0	12.5	5.5	2.0	2.5	0.75
OMEGA_HighAcc_4	PDB	0.0	37.5	44.0	10.5	5.0	2.5	3.0	0.72
iCon_HighAcc_4	CSD	1.2	76.6	18.5	2.9	0.6	0.2	0.2	0.40
OMEGA_HighAcc_4	CSD	0.4	75.1	21.6	2.5	0.2	0.2	0.2	0.40

**Table 6:** Percentage spreading of TC score values calculated for conformers of PDB and CSD compounds generated by iCon and OMEGA using three representative setting patterns.

**Settings pattern**	**Data set**	**>1.95**	**>1.75**	**>1.50**	**>1.25**	**>1.0**	**>0.75**	**< 1.0**	**Mean**
									**TC score**
iCon_LowAcc_4	PDB	1.0	21.0	29.5	20.5	16.0	11.0	12.0	1.45
OMEGA_LowAcc_4	PDB	1.0	24.0	33.0	18.0	11.0	11.0	13.0	1.49
iCon_LowAcc_4	CSD	7.5	48.2	27.0	12.9	3.7	0.6	0.6	1.72
OMEGA_LowAcc_4	CSD	6.7	49.3	24.3	14.6	4.4	0.8	0.8	1.71
iCon_MedAcc_2	PDB	1.0	24.5	32.5	23.5	11.5	7.0	7.0	1.51
OMEGA_MedAcc_2	PDB	1.0	29.0	38.5	15.5	11.0	4.0	5.0	1.57
iCon_MedAcc_2	CSD	7.5	54.7	26.2	9.4	2.1	0.2	0.2	1.75
OMEGA_MedAcc_2	CSD	6.9	56.3	25.4	8.3	2.7	0.4	0.4	1.75
iCon_HighAcc_4	PDB	3.0	31.5	26.5	23.0	9.0	6.5	7.0	1.55
OMEGA_HighAcc_4	PDB	3.5	41.5	23.0	16.0	10.5	4.5	5.5	1.60
iCon_HighAcc_4	CSD	14.1	57.6	19.1	7.9	1.2	0.0	0.0	1.80
OMEGA_HighAcc_4	CSD	16.4	54.9	20.4	5.8	2.3	0.2	0.2	1.80

The analysis of the results reported in Tables 5, 6 clearly demonstrates a high performance for both programs using the three setting patterns considered, since more than 50% of the generated conformational ensembles produced a very good matching with the ligand reference poses, giving TC scores ≥1.50 and RMSD values ≤ 1.0 Å. Precisely, as the PDB data set is concerned, for a minimum of 51.5% up to 68% of the ligands, a TC score above 1.50 was obtained for iCon_*LowAcc_4* and OMEGA_*HighAcc_4*, respectively (Table 6), while the percentage of molecules showing RMSD values below 1.0 Å (Table 5) ranged from 69.5 up to 81.5%. Compared to iCon, OMEGA always gave better results for the PDB data set in terms of TC score, consistently with what observed in the overall comparison of the two programs' performance. OMEGA produced 58.0–68.0% of conformational ensembles with TC scores ≥ 1.50, on average 8% more than iCon (51.5–61.0%), for which a shift toward lower TC score values was observed. Moreover, OMEGA yielded 25.0% of ensembles with excellent fit (TC score ≥ 1.75) using *LowAcc_4* settings and 45.0% with *HighAcc_4*, whereas those obtained with iCon for the same settings were 22.0 and 34.5%, respectively. The RMSD values revealed a slightly different situation (Table 5). Not only the difference in the percentage of ensembles with RMSD ≤ 1.0 Å generated by iCon and OMEGA was marginal (69.5–79.5 and 73.0–81.5%, respectively) but iCon also produced a number of ensembles with RMSD ≤ 0.5 Å (31.5–39.5%) higher than that shown by OMEGA (25.0–39.0%). In particular, with *LowAcc_4* settings iCon produced 6.5% more excellent-fitting conformers with respect to OMEGA. Similar results were obtained by the analysis of *LowAcc_3, MedAcc_1*, and *HighAcc_3* setting patterns (see Supplementary Tables 1, 2). These results underline the complementarity of the two different metrics, which are based on two different methods of structure superposition and thus gave different results that seemed to be the more divergent the higher the dimensions and the conformational freedom of the considered compounds.

Concerning the CSD data set, a higher number of compounds with a very good matching between generated and experimental conformations was obtained by the two programs, with respect to the PDB ligands (consistent with the higher mean TC scores and lower mean RMSD values), but the results in terms of the two metrics were more similar to each other. For instance, the number of CSD compounds for which a TC score ≥ 1.50 and a RMSD ≤ 1.0 Å was obtained ranged from 80.2% (OMEGA_*LowAcc_4*) to 91.7% (OMEGA_*HighAcc_4*) and from 91.5% (iCon_*LowAcc_4*) to 97.1% (OMEGA_*HighAcc_4*), respectively. With *LowAcc_4* and *MedAcc_2* settings iCon generated a higher number of perfect fitting conformers with respect to OMEGA in terms of both metrics, with 0.8% of compounds showing a RMSD ≤ 0.1 Å and 7.5% presenting a TC score ≥ 1.95 (compared to 0.2% and 6.7–6.9%, respectively, as obtained for OMEGA), as well as a lower percentage of failures. For *HighAcc_4* settings, for which the two programs gave equal values of mean RMSD and TC scores, iCon produced less ensembles comprising perfect fitting conformers than OMEGA, in terms of TC score (14.1%, and 16.4% for OMEGA) but more in terms of RMSD (1.2 vs. 0.4%). For all these settings iCon showed a small enrichment in compounds with RMSD ≤ 0.5 Å (ranging from 61.8 to 77.8%) with respect to OMEGA (59.5–75.5%), but gave a marginally higher number of molecules with TC score ≥ 1.75 only with *HighAcc_4* settings (71.7, 71.3% reported for OMEGA).

Overall, the obtained data indicate that both software packages actually perform in a similar way, with OMEGA giving only slightly better results when a medium-to-high quality sampling of larger and more flexible compounds is carried out; also, these differences are mainly relative to the TC score.

#### Influence of rotors in RMSD and TC score values

**Figure 6:**
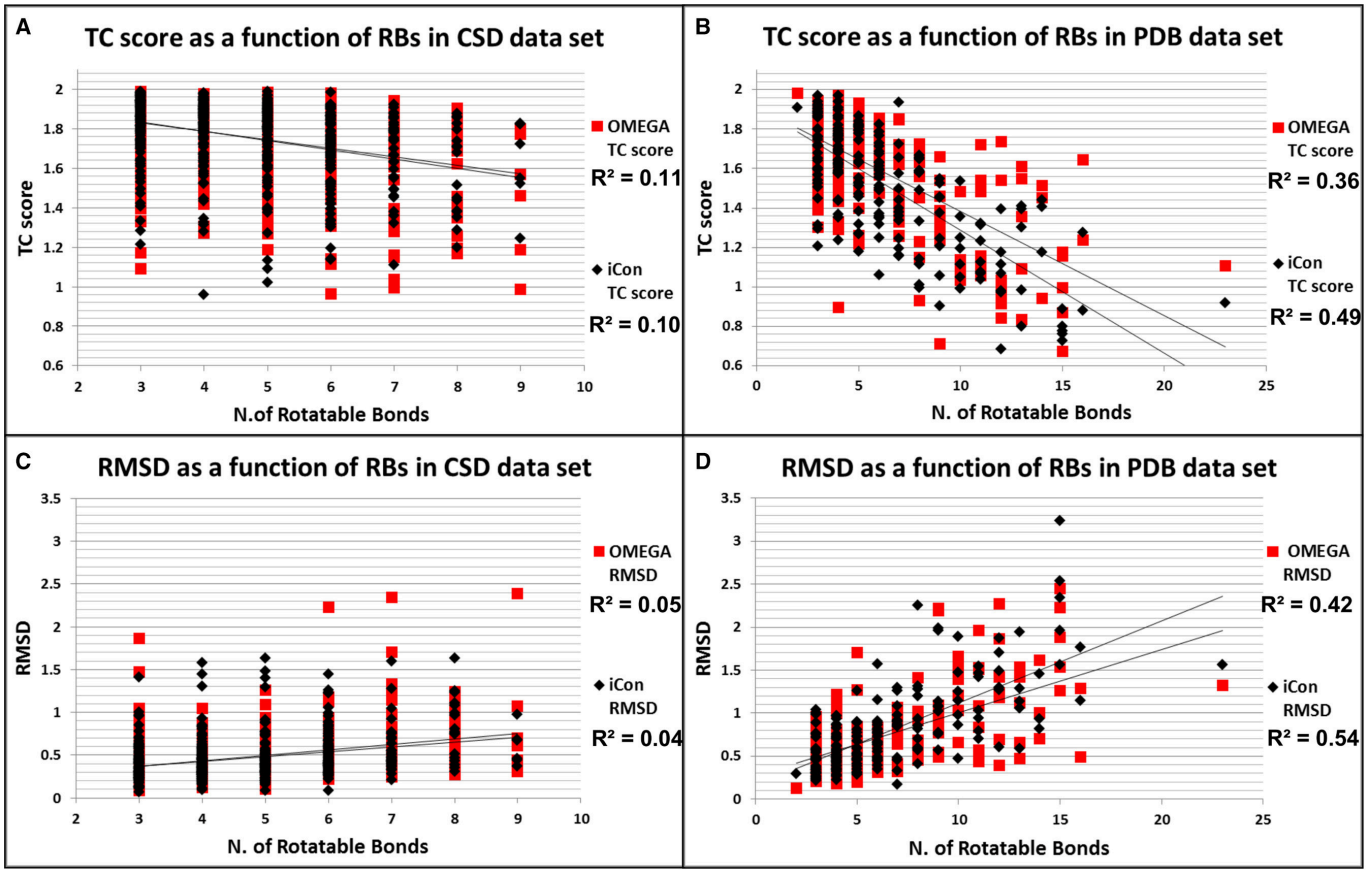
Distributions of TC score values as a function of the number of rotatable bonds for CSD **(A)** and PDB compounds **(B)**. Distributions of RMSD values as a function of the number of rotatable bonds for CSD **(C)** and PDB compounds **(D)**.

To better assess how the conformational freedom of the data set compounds influenced the performance of the two programs in reproducing experimental conformations, we plotted the obtained results in terms of RMSD and TC scores for PDB and CSD molecules by using *MedAcc_2* settings (as a reference setting) as a function of the number of rotors of the compounds (Figure 6). As expected, quite different trends were observed for the two data sets. For CSD molecules almost no correlation was found between the number RBs and the relative RMSD and TC score values given by iCon and OMEGA, which showed an almost identical distribution of the results with respect to both metrics (Figures 6A,C). For PDB ligands an appreciable correlation between conformational freedom and sampling performance was identified for both programs and particularly in terms of RMSD values, for which RBs/RMSDs correlation coefficients of 0.42 and 0.54 were calculated for OMEGA and iCon, respectively (Figure 6C). iCon's performance seems to be more influenced by the number of rotors compared to OMEGA, in accordance with what observed in the previous analyses. However, the difference in *R*^2^ values was quite small and the distribution of the results in terms of both metrics was pretty similar for the two programs, which again showed a comparable behavior.

#### Computational resources

**Figure 7:**
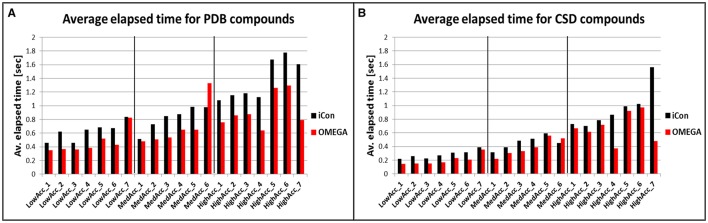
Average time required for the conformational sampling of PDB **(A)** and CDS **(B)** compounds with iCon and OMEGA, by using the different setting patterns. Black vertical lines are used to separate the three different groups of settings.

To compare iCon's efficiency in terms of computation time with OMEGA and to understand how it is affected by the different settings, we reported the average time required by the two programs for the conformational sampling of PDB and CSD compounds by using the various settings patterns. Both conformer generators proved to be fast, especially in the sampling of the CSD data set (Figure 7B), which required < 0.4 s per compound (s/cpd) for all the low accuracy settings and < 0.6 s/cpd for all the medium accuracy settings. OMEGA showed generally a better efficiency with respect to iCon, even though for this data set the differences in the average elapsed time were substantial only for *HighAcc_4* and *HighAcc_7* settings, where a RMSD cutoff for saving conformers of 0.2 Å was used. Using these two settings OMEGA was particularly fast (0.371 and 0.478 s/cpd, respectively) considering the elevated number of conformers generated. On the contrary, when a RMSD cutoff of 0.8 Å was used in *MedAcc_6* settings iCon was found to be faster than OMEGA (0.450 and 0.522 s/cpd, respectively) while for *LowAcc_7* settings the difference between the two programs was marginal. With the CSD data set, *LowAcc_4* and *MedAcc_2* confirmed to be efficient setting patterns for iCon (compared to the other low and medium accuracy settings), considering the performance in terms of mean RMSD and TC score with respect to the calculation time and the average NOC generated. The same can be said for *HighAcc_4* among the high accuracy settings, which proved to be particularly efficient also for OMEGA. In fact, OMEGA employed less than half of the sampling time required by iCon using these parameters and was faster even with respect to the *MedAcc_4-6* settings.

The sampling of the PDB data set took, in general, a longer time for both programs. This is in accordance to the higher conformational freedom of these ligands with respect to the CSD molecules. For this data set the gap between iCon and OMEGA was more evident, the latter being 26% faster, on average. Nevertheless, such a difference can be attributed to iCon's caching strategy, which was designed in order to allow a conformational sampling that is getting faster with a growing number of compounds in the database to be sampled. Precisely, the conformations generated by iCon for the compounds terminal fragments are continuously stored in a cache, thus having no necessity to be recalculated when the same fragments are encountered in further input compounds during the sampling process (see section Conformer Generation With iCon). However, the performance results clearly show that it might be worth thinking about changing the current caching strategy toward a prebuilt start fragment cache (like OMEGA has one) that is updated with newly encountered fragments. This would allow for overall faster calculations also for small compound libraries where the current caching strategy does not provide any significant speedup in the conformational sampling process. The influence of the various setting parameters on the efficiency of the two programs was much stronger with PDB data set (Figure 7A). OMEGA was remarkably affected by the *rms* parameter, showing again a faster sampling for *rms* = 0.2 Å (*HighAcc_4* and *HighAcc_7* settings) and a substantial increase in computation time when an *rms* value of 0.8 Å instead of 0.5 Å was used (e.g., *MedAcc_6* vs. *HighAcc_1*). On the contrary, this effect was not observed for iCon, which was appreciably faster than OMEGA for *MedAcc_6* settings and seemed to be mostly affected by the *e-window* value, in particular for the generation of medium- and small-sized conformer ensembles. With *LowAcc_1, LowAcc_3*, and *MedAcc_1* settings, for which an *e-window* value of 10 kcal/mol was used, iCon showed very similar calculation times (from 0.456 to 0.516 s/cpd) although the average NOC ranged from 17.5 to 102.2 compounds per ensemble, respectively (see also Figure 3). Similarly, in *LowAcc_2, LowAcc_4-6*, and *MedAcc_2* settings the *e-window* was set to 15 kcal/mol and the sampling time only ranged from 0.650 to 0.733 s/cpd even though the average NOC was raised from 22.2 to 114.4 compounds per ensemble. When larger ensembles were generated, the *e-window* seemed to have a smaller impact on iCon's efficiency compared to the other parameters. These results also showed that the improvement in iCon's performance obtained by increasing *e-window* of 5 kcal/mol was paid with an increase of computation time of nearly 40% the for PDB data set. This makes *LowAcc_4* and *MedAcc_2* settings not really convenient for the sampling of PDB-like molecules compared to the *LowAcc_3* and *MedAcc_1* settings, respectively. Anyway, *MedAcc_1* showed to be a very efficient setting for PDB ligands, requiring just a 12.7% longer sampling time than *LowAcc_3* but with much better results in terms of both RMSD and TC score values, making it suitable not just for medium-sized databases but also for large ones, despite the higher NOC generated. For the high accuracy settings, *HighAcc_3* seemed to have a good efficiency, giving results nearly as good as *HighAcc_5-6* but in less time (averagely −31.6%).

#### Notes on using the reproduction ability of crystallographic conformations as a performance measure

Before concluding the performance assessment of iCon it is worth mentioning the induced folding problem, i.e., the structural adaptation of the target protein to the ligand in order to form an optimal complex. The ligand-induced folding of the target receptor, which can be observed particularly in flexible protein such as tyrosine kinases after drug-target association, is a well-known issue in drug design (Fernández, 2016). Due to this effect, it is unlikely that the conformation of the target protein remains unchanged upon interaction with different ligands. Therefore, the target protein should not be considered as a rigid body in structure-based drug design studies: the flexibility of the corresponding target should be taken into account in combination with the conformational space of the ligand. However, the conformational flexibility of the protein is usually studied through computationally expensive molecular dynamic simulations which allow a thorough evaluation of the conformational motion of both ligand and protein at the same time. On the contrary, in docking studies the structure of the protein is normally treated as a rigid body allowing at most just a movement of residue side chains. Thus the conformational sampling of the docking algorithm only considers the ligand as flexible and largely neglects the adaption ability of the receptor. Moreover, in pharmacophore modeling and pharmacophore-based virtual screening, as well as in ligand-based similarity approaches, the protein structure is not even considered except for the generation of receptor-based pharmacophore models, and in this latter case only a single conformation of the protein is usually used. Therefore, all common conformer generators, especially those used for virtual screening purposes such as iCon and OMEGA, perform only the conformational sampling of small molecules in a way that is totally independent from the structure of any possible target protein. Indeed, there is no need to consider the conformational variability of the protein because it is intrinsically taken into account due to the fact that the output of the conformer generation is not a single conformer of a drug-like molecule but an ensemble of conformers that covers many structurally different protein conformations. For this reason, our performance assessment of iCon was only based on the reproduction of experimental structures of small molecules, a methodology that is widely used and reported in literature (Hawkins et al., 2010; Miteva et al., 2010; O'Boyle et al., 2011; Ebejer et al., 2012; Hawkins and Nicholls, 2012; Friedrich et al., 2017).

## Conclusions

In this study we report the algorithm of the novel conformer generator iCon implemented in LigandScout 4.0 and the assessment of its performance in comparison to OMEGA by using two different data sets of high-quality crystal structures from the PDB and CSD databases. We evaluated iCon's efficacy in reproducing the experimentally determined conformation of the test compounds in terms of RMSD and TC score values for 20 different setting patterns and we compared the results with those obtained with OMEGA using equivalent settings. The three parameters changed in these setting patterns showed to affect the size and the quality of the conformational ensembles generated by iCon for the two data sets in a different manner. The results indicate that in order to obtain an adequate sampling of the conformational space, a *max-num-conf* lower than 50 should be avoided, even for the generation of small ensembles. Moreover, an *e-window* value not lower 15 kcal/mol is recommended to improve iCon's performance, but this might be paid with an increase of computation time that might not be suitable for high-throughput conformational sampling. An *rms-thresh* value of 0.5 Å showed to be quite appropriate for all kind of conformational ensembles, even though some small adjustments based on the molecular properties of the sampled compounds can lead to better results. *LowAcc_3-4* and *MedAcc_1-2* settings proved to be good for a high-throughput and average quality sampling, while for a more thorough conformational analysis *HighAcc_3-4* settings represent a better choice.

Compared to OMEGA, iCon showed its best performance in the reproduction of crystallographic poses of less flexible molecules through small conformational ensembles, slightly outperforming OMEGA in the results obtained for CSD compounds with low accuracy settings. With the CSD data set, iCon yielded high quality results also when larger ensembles were generated, showing a lower or equal number of failures with respect to OMEGA for most of the setting patterns. Also, the spreading of RMSD and TC score values proved to be extremely similar. OMEGA is more effective in the sampling of ligands with higher conformational freedom, since with PDB data set it always produced better results than iCon, whose performance is more influenced by the number of rotors of the sampled compounds. However, the observed differences were still small, particularly when settings yielding small conformational ensembles were considered; also, such differences were primarily related to the TC scores. OMEGA proved to be always slightly faster than iCon, particularly in the conformer generation of PDB ligands but, on the basis of its algorithm, iCon's computation times decrease when larger databases are sampled. Moreover, iCon always showed to generate smaller conformational ensembles than OMEGA for equivalent settings, which can speed up any analysis based on iCon's conformational sampling, like pharmacophore modeling or virtual screening processes. Overall, the study herein reported proved that iCon represents a solid and well validated new conformer generator that comes free of additional charge with LigandScout 4.0 and is seamlessly integrated in all pharmacophore modeling and virtual screening related workflows of LigandScout. For a further improvement of iCon, the adoption of a different input molecule fragmentation and terminal fragment caching strategy is planned. This will not only speed up the conformer sampling process in general but will also lead to better results when it comes to the reproduction of bioactive conformations of larger and more flexible molecules.

## Author contributions

GP prepared the data sets, performed all computations, analyzed the results, and wrote the paper. TS implemented programs for data preparation and analysis, planned and supervised the study and contributed to writing the paper. TL contributed to writing the paper.

### Conflict of interest statement

The authors declare that the research was conducted in the absence of any commercial or financial relationships that could be construed as a potential conflict of interest.

## Supplementary material

The name of the 200 PDB structures from which the ligands used in this studies were extracted and their corresponding ligand codes. The codes of the 481 CSD compounds used in this studies. A figure showing the different average number of conformations generated by iCon and OMEGA for PDB and CSD data sets, using all the different setting patterns. A figure showing the average number of conformers generated by iCon as a function of the number of rotatable bonds in PDB and CSD compounds, using all the different setting patterns. Tables showing the percentage spreading of RMSD and TC score values calculated for conformers of PDB and CSD compounds generated by iCon and OMEGA using *LowAcc_3, MedAcc_1*, and *HighAcc_3* settings.

## Abbreviations

CAMD: computer-aided molecular design
CS: conformational sampling
CSD: Cambridge structural database
HA: heavy atom
MMFF94: Merck molecular force field
NOC: number of conformers
PDB: protein data bank
RB: rotatable bond
RMSD: root mean square deviation
TC: Tanimoto combo
VS: virtual screening.
