# Supplementary material for: Conformational Sampling of Small Molecules With iCon: Performance Assessment in Comparison With OMEGA
Source: Front Chem. 2018 Jun 19;6:229. doi: 10.3389/fchem.2018.00229 (PMC6018197; doi:10.3389/fchem.2018.00229)

Supplementary Material

Conformational Sampling of Small Molecules with iCon: Performance Assessment in Comparison with OMEGA

Giulio Poli, Thomas Seidel^*^, Thierry Langer

*** Correspondence:** Thomas Seidel: thomas.seidel@univie.ac.at

# Supplementary Data

## CSD Data Set Molecules

ABHYTZ

ABIMAZ

ABSBPP

ABVPRO

ABXBPC

ABZNPS

ACENHT

ACPIXZ

ACTHBZ

ADPRTR

AMOCMS10

AMPDAM

ANOPPS

ANTZOA

ANTZOB

AOPCHY

APRTAM

AURMIN

AZLNPR

BAFJEX10

BAFREF

BAFYOW

BAJZOB10

BATROD

BATXID

BAXOZO

BAZGAK

BBSPRT10

BEDLUR

BEHHOL10

BEKDIE

BELWOE

BENPRL

BESCEH

BETFAH

BEWLIY10

BEWLOE10

BEXVOP

BHMPET

BIBHID

BIBSEK

BSBTHP

BTHYDX

BUDCUY

BUFXOP

BUHNIB

BUNFUL

BUSNIM

BUTDID

BUVYIA

BUYZIE

BZAPCX10

BZAPUC20

CABCUD

CAFGIZ

CAGXEN

CALLEG

CANPUC

CARNID

CATXIE

CAVNUI

CAXBEI

CBMHZP

CBSTHP

CBZHYX

CDBMPI10

CEJTIU

CEKNOV

CELDEC

CELXOG

CENNIS

CETZOQ

CEVTUS

CEWFUF

CFBPBI

CIBPEI

CIBPOS

CIDSIR

CIHFAA

CIHFEE

CIHJAE

CIJFEG

CIJVAS

CIPXEE

CIXFIY

CIXTOS

CLPHAC

CLPNXA

CLPSAM

CLPTBU

CMANPQ

CMAPTX

CMPEPI

CMSMOC

CMXPMB

CMXPYO

CNBPCT

CNINDO

COCHOL

CODYUP10

COFHIO10

COKBOT

COKSUQ

COKTAX

COTXUE

COVXIU

COYHUT10

COYPIP

COYTOZ

COYVIV

COZFOM

CPIPLA

CPPHAM

CPQZOL

CPYPTZ

CUHNEY10

CUJSAB

CUNTIO

CUSLUX

CUWRIV

CYCLIZ10

DABGUI

DADDUH

DAFTOT

DAFVUB

DAHNIJ

DAKGEB

DANNEL

DAWRAU

DAXKAO

DCLPET

DCTXAN

DEBBER

DEBBOB

DEBFAR

DECZIU

DENBUT

DENDAC

DERZAB

DETGIS

DIAVER

DIGSIV

DIPJAN10

DISHOC

DIWPUU

DIWWUB

DIZREJ

DIZWAK

DOHREX

DOLJIX

DOPXIP

DOTFEX

DOXANN

DUDNUL

DUDPAT

DUHSUU

DUMTAG

DUPKII

DUXYOK

DUZDAD

DUZRAR

EABRUU

EBTNIC

ECBHMP

EHMPYX10

EITDZL

EMZPPO

EPCMPO

EXOADN

FABSOQ10

FABWUA10

FACCER10

FAGHIE

FAHTUD

FAHXIV

FAJVAN

FANPUF

FANRER

FAVYEG

FAWREA

FAXPUP

FBPAZD

FECPUY

FEFPOV

FEKYEZ

FEMMAL

FEVZUB

FEWBAK

FEYJEY

FEYKUP

FHFPBY

FIBREN

FIJJUD

FIVPUV

FIWLUS

FIXNUV

FLPNTX10

FOBZIF

FOGWAZ

FOJZUZ

FOLMEY

FOLYIO

FOMDUG

FOPMIG

FOWPOW

FOYLIO

FPAMCA

FUCFIS

FUDVUV

FUGSIJ

FUHHAR

FUHLID

FUJRUX

FUMBOE

FUMZAO

FUNXUH

FUNYAO

FUPFIF

FURACM

FURKEI

FURSEM01

FUSZOI

GAKWIY

GALMOV

GALSEM

GANFIK

GASPUL

GASXED

GATPOG

GAVMIZ

GEBJOM

GEHKIN

GEJJUA

GEJLAI

GEKWAU

GEWSOQ

GIFZAW

GIHKAJ

GIHTUM

GIHWUP

GIKJOZ

GINKUJ

HADLAZ

HALDOL

HALJOT

HALOPE

HALSES

HASCOT

HATKES

HAVJET

HAVLUL

HAVRUR

HBFRAN

HECHEC

HEHVOF

HEJTAR

HEKROE

HEKZAY

HEMLOA

HEMWUR

HEMXIG

HEPGAK

HEPKUI

HEPYAC

HEVJAT

HEVRIJ

HEVXEL

HEXFEV

HEXYIS

JOTDAX

JOXFIL

JUBGES

JUBJOF

JUDLUP

JUDWEK

JUHHEZ

JUKZUK

JUVPAR

KABJOM

KACKII

KAMYAY

KAVLIC

KAVNOK

KAXXAI

KEBVES

KECDUR

KEJBEG

KEJGEL

KEMHOZ

KICRIX

KIFZAA

KIHWAZ

KIHWED

KINJIA

KIXLOS

KOFKUL

KOFLIA

KOFLOG

KOFMIB

KOJMEB

KOKBIV

KOTGIJ

KUBHEU

KUFPIK

KUHNUW

LABLOP

LACCEX

LACMAD

LACPAG

LADTOZ

LANXED

LAWJEY

LAXWEM

LEDWAS

LEKMET

LELROJ

LEMXIK

LERKAU

LETBAN

LETBUH

LEVTOV

LEZGOM

LIKBUC

LIKGIV

LIKMEX

LINLAV

MAMNAQ

MATSTA10

MBZTZT10

MICONZ

MINTSA

MLDPHE10

MOPBZA

MRMPYR

MTBPNP

MVERIQ

NADYIA

NADZIB

NBPENC

NFCSEY

PACVUK

PAPPHI

PBBSHD

PBYXCH

PCPTZA

PEBHAF

PEKCEO

PEPHEX

PEXFED

PEXJEH

PIBSXM

PIDSIE

PIDWOO

PIKDIW

PILCOC

PINSAG

PKOJSI

PMAANO01

PMANAQ

PMBSAN10

PMCPRC10

PMEPEN

PMPAIN

PMPBTZ

PMTSZD

PNBPIP

PNPAUR

PONXUL

POPDAZ

PTHPAL

PXBVCP10

PXEZSD

PXMPEN

PYMSBZ

QQQHDG10

SADXIE

SAGMUI

SAGWOM

SAVXOC

SAVYET

SAVZEU

SAWZUL

SEFLIY

SEMDET

SEMXEN

SEYMIS

SEYPUH

SFDMOX

SIFJIA

SIHDIW

SIHGIZ

SIHVUA

SILTUC

SINKUV

SISYIC

SIZDOU

SLFNMB04

SLFNMF02

SOPNAM

SOPSIZ

SORCOR

SOWCOW

SPIRIL

SUKPET

SURREC

TAJSEC

TANNOL

TAPBZO

TAPSOS

TAPZEP

TAZPUF

TAZXAT

TECSUP

TEDBUZ

TEKBIU

TPHPRO

TSPOXZ

VADTAV

VAMBOA

VAMCUH

VAYXOI

VEMCIZ

VENRUB

VEXROF

VEYRAS

VIBNID

VIBWIM

VIMYUL

VIPHOR

VOPDIN

VORBAF

VORDAH

VORJER

VOWXEK

VOXHAR

VOXMOK

VOXNOL

VUGDEG

VURTOR

VUSKID

WAMZAL

WASTEP

WAVTIW

WEBCOV

WELGAV

WESFOP

WESZOJ

WEVTIA

WEXBEG

WIDDIW

WIFREI

WIKJOP

YABKOB

YAGWOS

YAHBOY

YAHFAO

YAYDEH

YAZBAC

YAZHUC

YEMNEJ

YENLEI

YESPIV

YEYVED

YICPIJ

YIHJAA

YILDOM

YINDII

YITLES

YITZUW

YOPRAW

YOPWOP

YOSMOI

YOWVOV

YOWYAK

YOXDEU

YUKKUK

YUKNAT

YULXIM

YULZOU

YUYJEH

ZZBUFXOP

## PDB Data Set Molecules

1b59 OVA

1b6j PI1

1b6k PI5

1b6l PI4

1b6m PI6

1bhx R56

1br6 PT1

1bto SSB

1c1u BAI

1c3i TR1

1cbs REA

1cgz STL

1cvu ACD

1cw2 HSP

1d09 PAL

1d3g BRE

1d3h A26

1d4i BEG

1d8d FII

1db1 VDX

1dfo FFO

1e2m HPT

1e6y TP7

1ee2 CHD

1eve E20

1exa 394

1ezf IN0

1f06 2NP

1f0t PR1

1f0u RPR

1f8b DAN

1fbl HTA

1fm9 570

1g2k NM1

1g4s TPS

1g6c TZP

1g9v RQ3

1gj6 132

1gja 135

1gjd 136

1gs5 NLG

1gz8 MBP

1h1p CMG

1h1s 4SP

1h2k OGA

1h6h PIB

1hvy D16

1hw8 114

1hwi 115

1hwk 117

1hyo HBU

1ig3 VIB

1is0 PTC

1iy7 CXA

1j3f CZM

1jd0 AZM

1k1j FD2

1k2t PTU

1k2u TFM

1k3u IAD

1ke5 LS1

1kz8 PFE

1kzk JE2

1l2s STC

1l7f BCZ

1lpm MPA

1lww PED

1m2x MCO

1m5b BN1

1m5d BRH

1m5f AM1

1meh MOA

1mmv 3AR

1mts BX3

1mtw DX9

1mzc BNE

1n2j PAF

1n2v BDI

1njs KEU

1nnk CE2

1nqu RDL

1nqv LMZ

1o2q 991

1o2y 847

1o3m 785

1ofd AKG

1oiu N76

1oks NHE

1opk P16

1p1o QUS

1pa9 CSN

1pck PEZ

1q1g MTI

1q6r LX1

1q72 COC

1q91 DPB

1q92 DRM

1qin GIP

1qxw M1C

1qy5 NEC

1r34 LKS

1r4p 1PS

1r55 97

1r6w 164

1rl4 BRR

1ros DEO

1s19 MC9

1s63 778

1s8j RET

1sa4 JAN

1sn5 T3

1so3 TX4

1sq5 PAU

1sr7 MOF

1syh CPW

1t32 OHH

1t40 ID5

1t46 STI

1toj HCI

1uf5 CDT

1uf7 CDV

1uf8 ING

1urw I1P

1utt CP8

1uu9 BI3

1uwc FER

1uy8 PU5

1v2k ZEN

1v2n BBA

1v97 MTE

1w1q ZIP

1w4l GL8

1w5v BE3

1w7x 413

1w9u RIG

1wvj IBC

1xap TTB

1xge NCD

1xm6 5RM

1xn0 ROL

1xom CIO

1xon PIL

1xoq ROF

1xp0 VDN

1xqc AEJ

1y6b AAX

1y6q TDI

1yc1 4BC

1yc4 43P

1yvx IPC

1z1r HBH

1z95 198

1zhy CLR

1zvx FIN

1zz2 B11

2aj8 SC3

2aw1 COX

2ayr L4G

2b7d C1B

2bgd T1D

2brc CT5

2btr U73

2bts U32

2byh 2D7

2byi 2DD

2c4w GAJ

2c92 TP6

2cbs R13

2cf9 348

2cji GSK

2evc FC3

2ewb ZED

2f14 FL1

2f34 UBA

2f4j VX6

2f6v SK2

2f70 UN6

2f71 UN7

2f7p 2SK

2flb 6NH

2fm0 M98

2fqt H1D

2fwz H71

2fyv W72

2g8n F83

2ggd S3P

2gss EAA

2h03 3UN

2h42 VIA

2hb1 512

2hd6 BOS

2i0a MUI

2iko 7IG

2iuz D1H

2izl IMI

2j34 GS6

2j47 GDV

2j4i GSJ

3cbs R12

6prc CEB

# Supplementary Figures and Tables

## Supplementary Figures


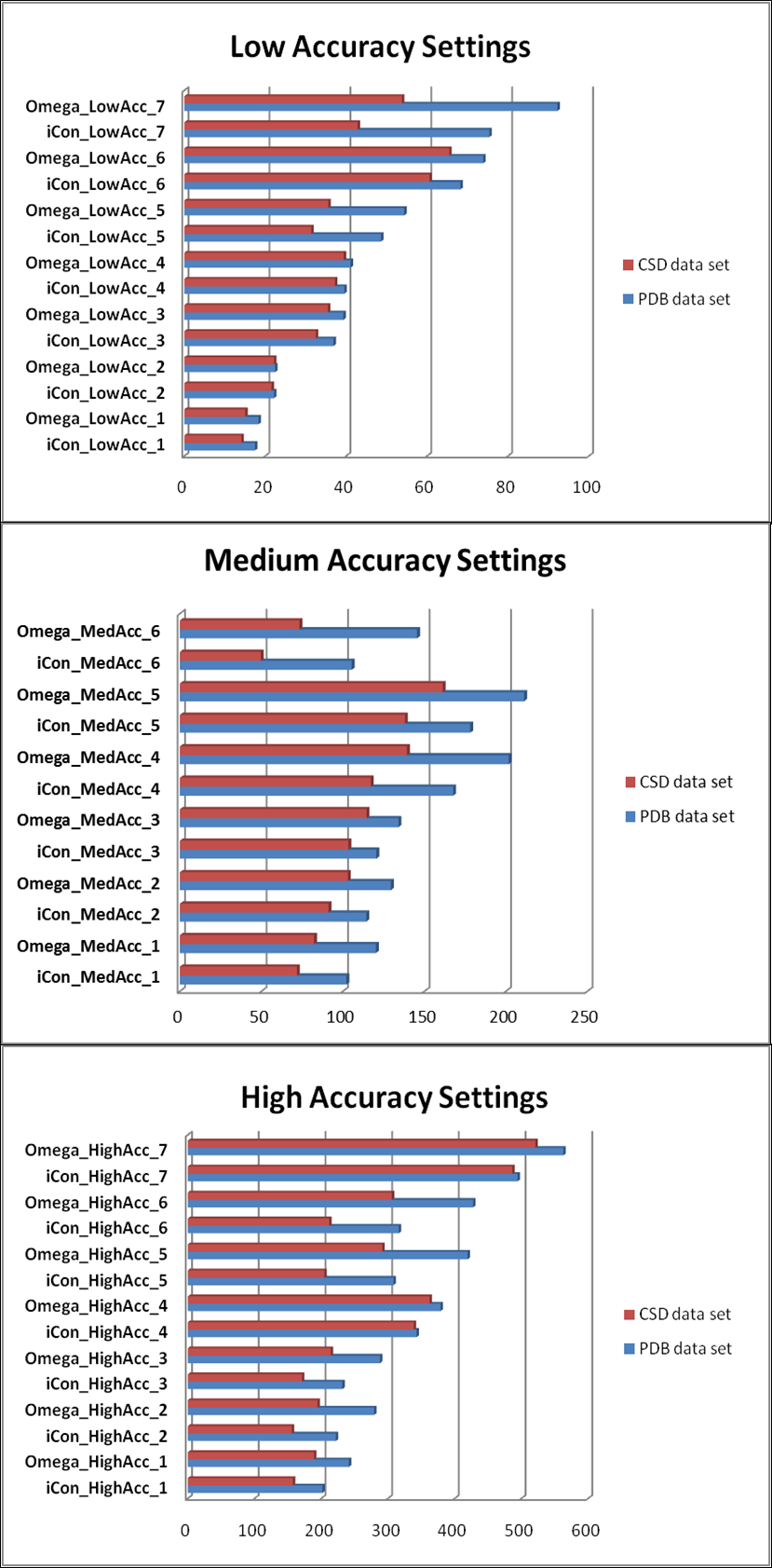


**Supplementary Figure 1.** Number of conformer (NOC) generated by iCon and OMEGA for PDB data set (blue bars) and CSD data set compounds (red bars) with low, medium and high accuracy settings.


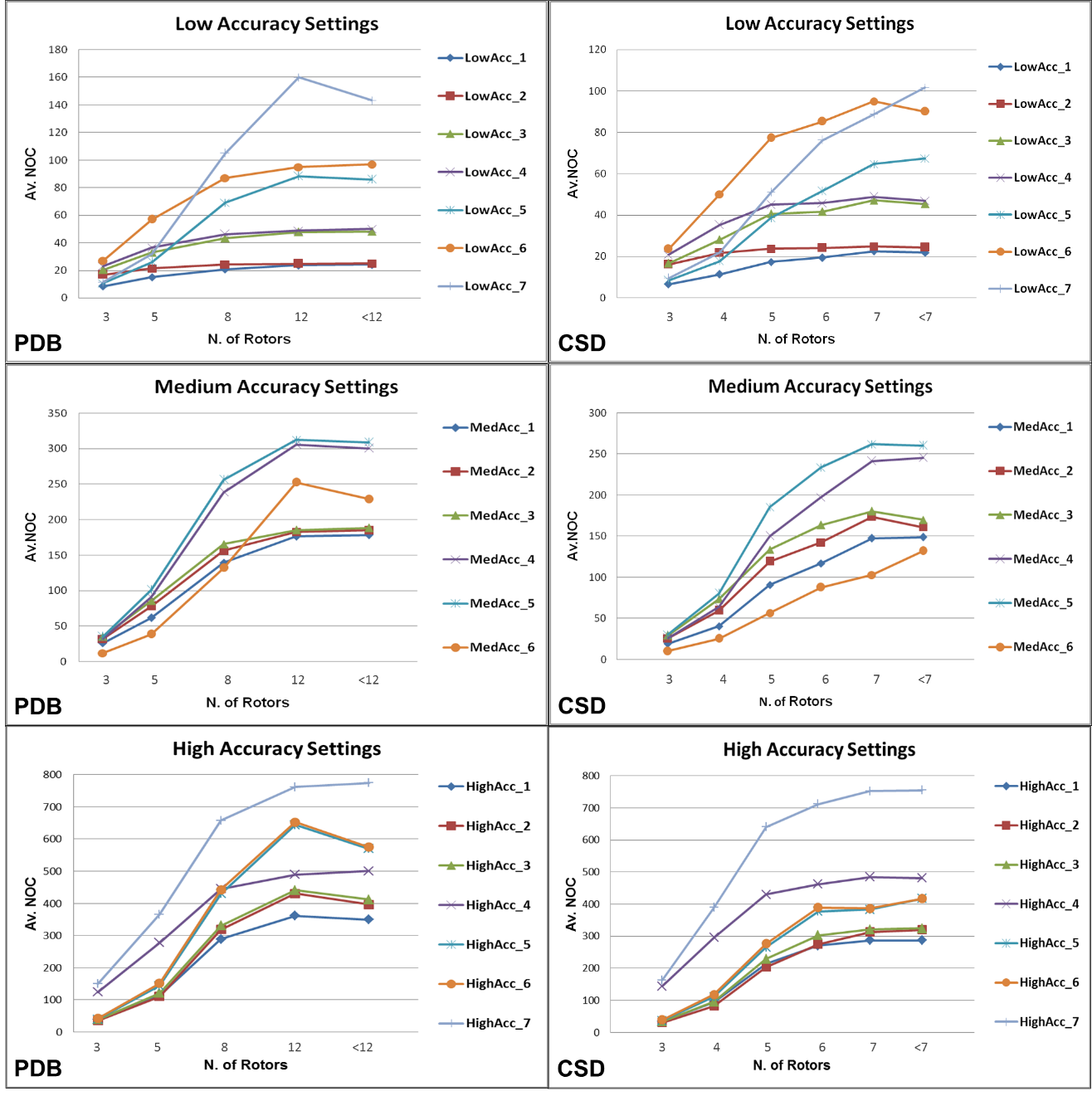


**Supplementary Figure 2.** Average NOC generated by iCon as a function of the number of rotable bonds for PDB and CSD compounds for Low, Medium and High Accuracy settings. Due to the different rotor distribution in PDB and CSD molecules, different scales have been considered for the two data sets.

## Supplementary Tables

**Supplementary Table 1.** Percentage spreading of RMSD values calculated of conformers of PDB and CSD compounds generated by iCon and OMEGA using LowAcc_3, MedAcc_1 and HighAcc_3 settings.


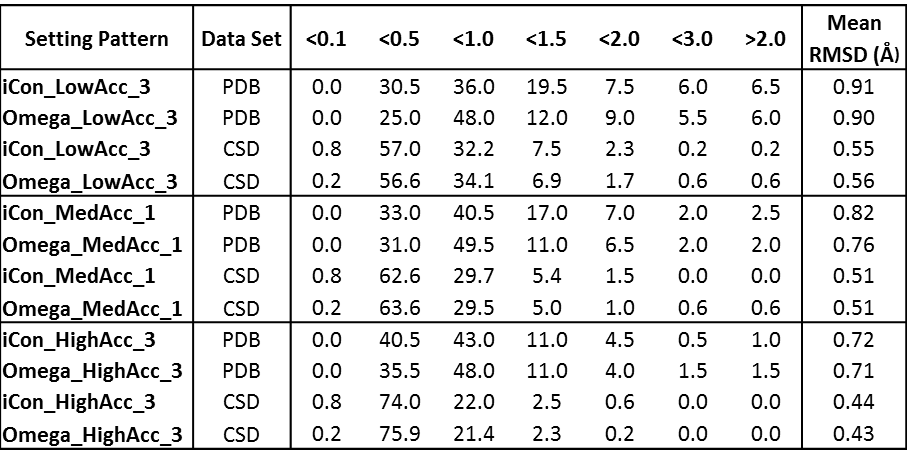


**Supplementary Table 2.** Percentage spreading of TC score values calculated for conformers of PDB and CSD compounds generated by iCon and OMEGA using LowAcc_3, MedAcc_1 and HighAcc_3 settings.


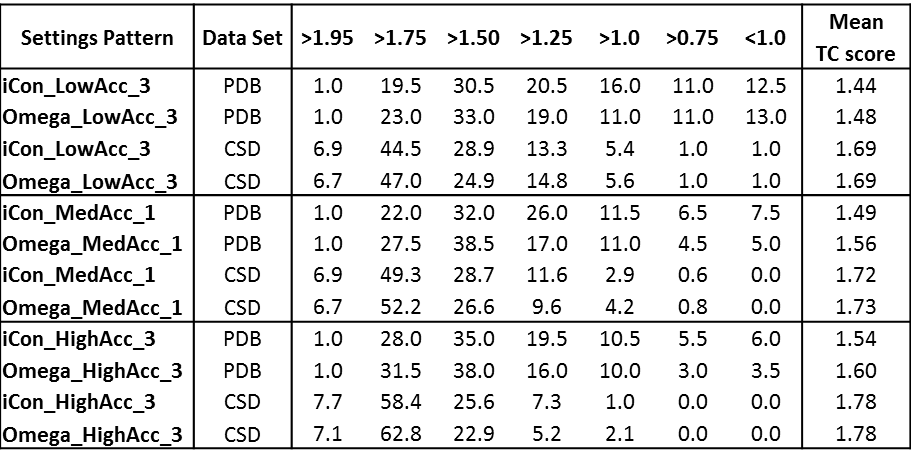

Supplement: Supplementary file 1 [file Data_Sheet_1.docx]
